# Supplementary material for: The lytic polysaccharide monooxygenase CbpD promotes Pseudomonas aeruginosa virulence in systemic infection
Source: Nat Commun. 2021 Feb 23;12:1230. doi: 10.1038/s41467-021-21473-0 (PMC7902821; doi:10.1038/s41467-021-21473-0)
Supplement: Supplementary file 1 — Supplementary Information [file 41467_2021_21473_MOESM1_ESM.pdf]

Supplementary Information

The lytic polysaccharide monooxygenase CbpD promotes *Pseudomonas aeruginosa* virulence in systemic infection

Fatemeh Askarian[1*], Satoshi Uchiyama[2‡], Helen Masson[3‡], Henrik Vinther Sørensen[4], Ole Golten[1], Anne Cathrine Bunæs[1], Sophanit Mekasha[1], Åsmund Kjendseth Røhr[1], Eirik Kommedal[1], Judith Anita Ludviksen[5], Magnus Ø. Arntzen[1], Benjamin Schmidt[2], Raymond H. Zurich[2], Nina M. van Sorge[6,7,8], Vincent G. H. Eijsink[1], Ute Krengel[4], Tom Eirik Mollnes[5,9,10,11], Nathan E. Lewis[2,3,12], Victor Nizet[2,13*] & Gustav Vaaje-Kolstad[1*]

[1]Faculty of Chemistry, Biotechnology and Food Science, Norwegian University of Life Sciences (NMBU), 1432 Ås, Norway. [2]Division of Host-Microbe Systems & Therapeutics, Department of Pediatrics, UC San Diego, La Jolla, CA, USA. [3]Department of Pediatrics, University of California, San Diego, School of Medicine, La Jolla, CA, USA. [4]Department of Chemistry, University of Oslo, P.O. Box 1033 Blindern, NO-0315 Oslo, Norway. [5]Research Laboratory, Nordland Hospital, Bodø, Norway. [6]Department of Medical Microbiology, University Medical Center Utrecht, Utrecht University, Utrecht, The Netherlands. [7]Department of Medical Microbiology and Infection Prevention, Amsterdam University Medical Center, University of Amsterdam, Amsterdam, The Netherlands. [8] Netherlands Reference Laboratory for Bacterial Meningitis, Amsterdam University Medical Center, Amsterdam, The Netherlands. [9]K.G. Jebsen TREC, Faculty of Health Sciences, UiT- The Arctic University of Norway, Tromsø, Norway. [10]Department of Immunology, Oslo University Hospital, and K.G. Jebsen IRC, University of Oslo, Oslo, Norway. [11]Center of Molecular Inflammation Research, Norwegian University of Science and Technology, Trondheim, Norway. [12]Novo Nordisk Foundation Center for Biosustainability at UC San Diego, University of California, San Diego, School of Medicine, La Jolla, CA, USA.[13]Skaggs School of Pharmacy and Pharmaceutical Sciences, UC San Diego, La Jolla, CA, USA.

‡These authors contributed equally to this work.
*correspondence: gustav.vaaje-Kolstad@nmbu.no, vnizet@health.ucsd.edu, fatemeh.askarian@nmbu.no

This PDF file includes:
Supplementary Results
Supplementary Tables 1-5
Supplementary Figures 1-17
Supplementary References

Other supplementary materials for this manuscript include the following:
Supplementary Data 1-11

SUPPLEMENTARY RESULTS

CbpD has an extended monomeric, tri-modular structure

Examination of the CbpD sequence in 93 whole genome sequenced clinical and non-clinical/environmental PA isolates extracted by Diamond BLAST (https://www.pseudomonas.com), revealed the presence of the CbpD encoding gene in all strains, with only minor variations in sequence. A multiple sequence alignment (ClustalW) of CbpD variants from representative isolates revealed no amino acid substitutions among essential LMPO active site residues (**Supplementary Fig. 1**). CbpD and orthologs consist of three major domains, an LPMO domain (AA10-type), preceded N-terminally by a signal peptide, in the middle a module with unknown function (Module X; or short MX), and a C-terminal family 73 carbohydrate binding domain (CBM73). These domains are connected with flexible linkers, of which in particular the linker connecting MX and CBM73 contains a large number of glycine residues **(Figs. 1a and Supplementary Fig. 1).**

The most similar homologous sequence to CbpD with a known 3D structure is that of *Vibrio cholerae* colonization factor GbpA (Q9KLD5, PDB ID:2XWX), a four-domain protein, with 32% sequence identity over the two first modules (AA10 + MX) and 46% sequence identity between the C-terminal modules (GbpA has an extra domain that is inserted between the MX and CBM domains). Lacking a crystal structure of CbpD, we built high-quality models of the AA10 and MX modules based on crystal structures of proteins displaying high sequence identity with these domains (**Table 3**). A satisfactory model of the CBM73 module, which resembles CMB5/12 domains, was also built (with P- and Score-values only slightly deviating from the recommended threshold values; **Table 3**). Templates for the third module included CBM12 (PDB ID: 4TX8 and 1ED7) and CBM5 (PDB ID: 2D49) domains from the CAZy database [1], which are more distantly related to CBM73 compared to CMB5/12 (**Supplementary Fig. 5a-b**). Note that two adjacent cysteine side chains were observed in the initial model of CbpD CBM73 (Cys333 and Cys342), which were modeled as a disulfide bridge in the subsequent MD simulations. These two cysteine residues are conserved in CBM73 sequences.

A final model that included all three folded modules and the two predicted linker regions L1 and L2 was used as an initial model for MD simulations. Intriguingly, natural CbpD carries post-translational modifications, including multiple phosphorylations [2, 3, 4] (**Fig. 1a-b**), and we were curious about what effect modifications of the abundant phosphorylation sites could have on the solution structure of the protein. Phosphate groups covalently attached to Ser, Thr or Tyr residues at protein surfaces may have a net charge of -1 or -2, depending on the pH. Thus, we prepared a model of CbpD that had no modifications (no-PTM) and two models that were fully phosphorylated, PTM-1 and PTM-2, where each phosphate group had a net charge of -1 and -2, respectively. When analyzing the structures that had been collected throughout 525 ns MD-simulations, the three models varying in PTMs displayed very different behavior. The model with no PTMs displayed a preference for shorter AA10, MX and CBM73 distances compared to PTM-1 and PTM-2 (**Supplementary Fig. 3a**). This finding indicates that the negatively charged PTMs on the AA10 and MX domains lead to domain separation in space due to electrostatic repulsion interactions. The overall shape of CbpD in solution is indicated by the radius of gyration of the protein. The data presented in **Supplementary Fig. 3b** indicates that negatively charged PTMs lead to a less densely packed three-domain structure of CbpD in solution, reflected in a higher radius of gyration.

The phosphorylation sites Y65, S195 and S197 are exposed on what can be considered the substrate-binding surface of the LPMO domain. When phosphorylated, these groups will most likely promote or prevent interactions with substrate and, thus, regulate the catalytic activity of the enzyme. The phosphorylation site at S197 is very close to the second sphere residue E199 that is part of the LPMO active site. A phosphate group at this site will most likely impact O_2 and H_2O_2 activation in the active site, and one could speculate that the redox potential of the Cu(I)/Cu(II) pair of the active site may be altered.

To obtain experimental data on the potential flexibility of CbpD, the solution structure of full-length CbpD was analyzed using Small Angle X-ray Scattering (SAXS) (**Figs. 1c and Supplementary Fig. 4**; SASBDB ID: SASDK42). Recombinantly produced full-length $rCbpD_{EC}$ was measured at 20 °C (**Supplementary Fig. 4a and 4b**). SAXS data and analysis are summarized in **Supplementary Table 2**. Both the forward scattering, obtained from the Guinier analysis (**Supplementary Fig. 4c**), and the Porod volume (**Supplementary Fig. 4e**) are consistent with a monomeric protein. The large radius of gyration of 32.16 +/- 0.11 Å, which approximately matches the predicted value (**Supplementary Fig. 3b**), and the shape of the pair-distance distribution curve (**Supplementary Fig. 4d**), both indicate an elongated protein shape (**Fig. 1c**). The maximum diameter of the protein was estimated to be approximately 120 Å. We plotted the data in a dimensionless Kratky plot (**Supplementary Fig. 4f**), giving a bell-shaped curve, consistent with a folded protein, but with a maximum at qRg = 3, which is higher than the maximum of qRg = $\sqrt{3}$ for completely globular proteins [5]. This indicated that the protein

is folded but has flexible regions. We generated 20 low-resolution models, which were averaged and refined to obtain one representative low-resolution model (**Fig. 1c**). CbpD has highly flexible regions, especially the L2 linker, which significantly limits the level of detail provided by the low-resolution model. It was therefore at first challenging to estimate which end of the molecular envelope belonged to the larger AA10 domain, and which to the CBM73. However, superimposing the atomistic CbpD Pepsi-SAXS model ($\chi 2$ = 2.35) onto the low-resolution *ab initio* model envelope resulted in a good fit (Fig. 1c), giving high confidence in the model. Comparison of the SAXS data collected at the synchrotron (ESRF) to the data collected on our home source at 24 °C (SASDJQ5) and 37 °C (SASDJR5) showed that the CbpD solution structure is consistent over this temperature range. The elongated shape of the CbpD model resembles the solution structure of the homologous GbpA, which was determined with SAXS by Wong *et al.* [6].

Transcription of *lpmO* genes is sensitive to environmental variations

In experiments complementary to the *cbpD* transcript analysis (Fig. 2a), expression of the LPMO-encoding gene in *Vibrio anguillarum* NB10 ($lpmO_{VA:}$ VANGNB10_cII0922c), a fish pathogen, and the LPMO-encoding gene in *E. faecalis* V583 ($lpmO_{EF:}$ EF_0362), a Gram-positive human pathogen, were also investigated in host-mimicking conditions. In contrast to *cbpD*, $lpmO_{VA}$ was present in low abundance during growth in minimal medium specific for *V. anguillarum* ($M9_{VA}$) in the mid-exponential phase, but its expression was ~ 19-fold higher in the presence of 10% normal salmon serum (SS) (**Supplementary Fig. 8a**). For $lpmO_{EF,}$ the transcript abundance during growth in minimal medium specific for *E. faecalis* ($M9_{EF}$) and in $M9_{EF}$ that was supplemented with 10% NHS and bacteriologic medium (BHI) (**Supplementary Fig. 8b**) followed the same trends as for *cbpD* (**Fig. 2a**).

CbpD is not cytotoxic and shows no binding to selected cell surface receptors

The putative cytotoxic effect of CbpD towards host immune cells was examined by assessing the response of THP-1 and differentiated HL-60 cells to $rCbpD_{PA}$. The recombinant protein did not induce major cytotoxicity at the examined concentrations up to as long as 24 h post-treatment (**Supplementary Fig. 11b**). Since the role of CbpD in PA infection was revealed using whole human blood, potential interaction of the LPMO on neutrophils were investigated. The resulting data showed that binding of antibodies directed against an array of cell-surface receptors associated with the innate immune function was not affected by rCbpD (**Supplementary Fig. 11e**), indicating that CbpD does not interact with the receptors.

Deletion of *cbpD* influences the performance of the terminal complement cascade

The production of anaphylatoxin C5a is mediated through cleavage of the C5 molecule by C5 convertase and represents a hallmark of terminal complement cascade activation. The influence of CbpD on the generation of C5a (**Supplementary Fig. 11f**) could indicate a role of the LPMO in modifying the activity of the complement cascade. The Wielisa®Complement system screening was used to evaluate the integrity of the classical (CP) and alternative pathways (AP) in the presence of $rCbpD_{PA}$. This assay is designed to quantify the assembly of C5b-9 neoantigen that is formed as an end product of complement system activation. Complement activity was mildly reduced upon supplementation of $rCbpD_{PA}$ to normal human serum, but not as efficiently as OmCI or the C5-depleted serum control (**Supplementary Fig. 11f**). The OmCI compound inhibits the terminal complement cascade, preventing cleavage of the C5 molecule [7].

Supplementary Table 1. Post-translational modifications on $rCbpD_{PA}$. The residues of $rCbpD_{PA}$ detected with either phosphorylation, methylation or acetylation when expressed in *Pse* PTMs were detected using proteomics with the software PEAKS 44 [8]. Only modified peptides falling within 0.1% FDR (-10logP > ~32.9; one-sided permutation test) and having an AScore > 20 were considered valid.

Residue*	$rCbpD_{PA}$	
	AScore	-10logP
T14p	26.65	36.71
S28p	28.35	51.66
S49p	177.39	66.78
K53m	77.52	58.25
K147m	131.93	52.86
K166m	93.59	34.94
R173m	60.15	71.11
K182m	38.15	45.59
K354a	1000.00	36.13
K359a	1000.00	36.13
K368a	1000.00	36.13

*p – phosphorylation, m – methylation, a – acetylation.

Supplementary Table 2. SAXS data collection and analysis (20 °C; ESRF BM29).

**(a) Sample details**	
	CbpD
Organism	*Pseudomonas aeruginosa*
Source (Invitrogen)	*E. coli* expression host (BL21 star)
Uniprot sequence ID (Residues in construct)	Q9I589 (26-389)
Extinction coefficient [A_{280}, 0.1% (=1 g/l)]	2.098
$\bar{v}$ from chemical composition ($cm^3\ g^{-1}$)	0.73
Particle contrast, $\Delta\bar{\rho}$ ($10^{10}\ cm^{-2}$)	3.01
*MM** from chemical constituents (kDa)	39.2
Protein concentration (mg/mL)	2.50, 1.25, 0.63 and 0.31 (data extrapolated to zero solute concentration)
Solvent	150 mM NaCl, 15 mM Tris-HCl, pH 7.5

**(b) SAXS data collection parameters**	
	CbpD
Instrument	BM29 with Pilatus2M detector at ESRF [9]
Wavelength (Å)	0.992
Beam size (µm)	700 X 700
Sample to detector distance (cm)	282.7
q measurement range ($Å^{-1}$)	0.00449 - 0.51787
Absolute scaling method	Milli-Q water standard measurement
Normalization	Transmitted intensities through semi-transparent beam-stop
Exposure time (h)	10 frames of 1 s
Radiation damage monitorization	Frame to frame evaluation and comparison with home-source data
Capillary size (mm)	1.8
Sample temperature	20 °C

**(c) Software employed for SAXS data reduction, analysis and interpretation**	
	CbpD
SAXS data reduction	BsxCuBE beamline software [9]
Extinction coefficient estimate	ProtParam [10]
Calculation of contrast and specific volume	*MULCh*1.1 [11]
Basic analysis	*PRIMUS* (*ATSAS*) [12, 13]
Shape reconstruction	*DAMMIF* [14]/*DAMAVER* [15]/*DAMMIN* [16]
Atomic structure modelling	*Pepsi-SAXS*[17]
Representation	PyMOL

**(d) Structural parameters**	
	CbpD
Guinier analysis	
I(0) (cm^{-1})	0.03086 ± 0.00007
R_g** (Å)	32.16 ± 0.11
q_{min} ($Å^{-1}$)	0.0137
qR_g max	1.29
R^2	0.987
MM from *I(0)* (kDa) (*ratio to predicted)*	38.7 (0.99)
P(r) analysis	
I(0) (cm^{-1})	0.0314 ± 0.0001
R_g (Å)	34.51 ± 0.1411
d_{max}*** (Å)	120
q range ($Å^{-1}$)	0.0137 - 0.2480 (0.1760)
χ^2	0.99
Total quality estimate from *PRIMUS*	0.78
MM from *I(0)* (kDa) (*ratio to predicted)*	39.4 (1.01)
Porod analysis	
Porod volume ($Å^3$)	64131
MM from Porod volume (kDa) (*ratio to predicted)*	40.1 (1.02)

**(e) Shape model-fitting results**	
	CbpD
DAMMIF	
q range ($Å^{-1}$)	0.0137 - 0.2480

Symmetry, anisotropy assumptions	*P*1, none
NSD (Standard deviation)	0.917 (0.192)
Constant adjustment	Skipped
Resolution (Å)	33 +/-3
MM from *DAMMIF* (kDa) (ratio to predicted)	30.1 (0.77)
χ^2	1.668-1.676
DAMAVER/*DAMMIN*	
*q r*ange	0.0137 - 0.2480
Symmetry, anisotropy assumptions	*P*1, none
χ^2	1.642
CorMap p-value	0.1403
Constant adjustment	Skipped

**(f) Atomistic modelling**	
	CbpD
Pepsi-SAXS	
Homology model	Raptor-X (link)
q range (Å^{-1})	0.00449 - 0.51787
Flexibility optimization	Enabled
Constant adjustment, allowed	-0.000127621
Rigid bodies, residues	AA1-115, AA192-292, AA307-367
χ^2	2.35

**(g) SASBDB ID**
SASDK42

*Molecular mass; **Radius of gyration; ****d_{max}: Maximum diameter

Supplementary Table 3. CbpD model quality*

Domain	P-value	Score	uGDT/GDT	Template PDB ID(s)**
AA10	1.6e-15	166	154/83	6IF7
MX	2.3e-05	97	83/77	2XWX, 2I1S
CBM73	5.2e-04	63	32/45	4TX8, 1ED7, 2D49, 5IN1, 4XZJ

* The P-value indicates the relative quality of the model where a low number indicates high model quality. Please see http://raptorx.uchicago.edu/documentation/#goto2 for further explanation of quality indicators P-value, Score and uGDT(GDT).

**The RaptorX program can use several PDB templates when available, and the PDB templates selected by RaptorX for each domain are listed in the fifth column (listed by ascending quality indicators). Quality indicators of the top scoring model for each domain are listed in columns 2-3.

Supplementary Table 4. Functional annotation of proteins grouping together in the cluster analysis (Figs. 2E and S8D) based on gene ontology (GO).

Cluster (Total number of protein)	GO term
I (n=168)	-*
II (n=369)	Oxidoreductase activity, acting on NAD(P)H, NAD(P) as acceptor<br>ATP-dependent peptidase activity<br>Glutamate-ammonia ligase activity
III (n=387)	Alkylbase DNA N-glycosylase activity<br>DNA-(apurinic or apyrimidinic site) endonuclease activity<br>Coproporphyrinogen oxidase activity<br>Ferroxidase activity<br>Glutamate 5-kinase activity<br>2-polyprenyl-6-methoxy-1,4-benzoquinone methyltransferase activity<br>Guanine deaminase activity<br>2-octaprenyl-6-methoxy-1,4-benzoquinone methylase activity<br>Amino acid binding
IV (n=594)	dUTP diphosphatase activity<br>Glucose-6-phosphate 1-epimerase activity<br>RNA polymerase binding
V (n=483)	Amino acid transmembrane transporter activity<br>ATPase-coupled amino acid transmembrane transporter activity<br>P-P-bond-hydrolysis-driven protein transmembrane transporter activity<br>Protein transmembrane transporter activity<br>Symporter activity<br>ATPase-coupled heme transmembrane transporter activity

*No functional annotation.

Supplementary Table 5. Primers and plasmids used in this study[*].

Plasmids/Primers	Properties/Sequence (5' - 3')	Use	Origin
PGM931	pGM931 pHERD20T derivative carrying araBp-tΩ region	Complementation	[18]
CbpD-KpnI-FW	GTTTCTCCATGGTACCGGCTGCGCTTCCTCAAGGGC	Plasmid construction for complementation	This study
CbpD-SfbI-RV	TAGAGTCGACCTGCAGGTTACAGCAGGGTCCAGGCG	Plasmid construction for complementation	This study
CbpD-SfbI-His$_6$-RV	AGAGTCGACCTGCAGGTCAATGGTGGTGATGATGGTG CGCCAGCAGGGTC	Plasmid construction for complementation	This study
CbpD-Out-FW	GCGGCCCTTTGCCTGCCTCTACCGG	Confirmation	This study
CbpD-int-RV	TGGCCTTGTAGACGAACTGGAA	Confirmation	This study
pGM-FW	TTTTCACCACCCCCTGACCGCGA	Confirmation	This study
rCbpD-**pNIC**-FW	**TTAAGAAGGAGATATACT**ATGAAACACTACTCAGCCA CCCT	Construction of different variants of His-tagged CbpD in *E. coli*	This Study
rCbpD-**pNIC**-RV	**AATGGTGGTGATGATGGTGCGC**CAGCAGGGTCCAGG CGTCCT	Construction of different variants of His-tagged CbpD in *E. coli*	This Study
rCbpD-AA10-**pNIC**-RV	**AATGGTGGTGATGATGGTGCGC**GCCGCTGAAGCTCA CGTCGATGCA	Construction of His-tagged AA10 module in *E. coli*	This study
rCbpD-MX/CBM73-**pNIC**-FW	**TTAAGAAGGAGATATACT**ATGGCCGTCGCCAACCCCT GGCAA	Construction of His-tagged MX+CBM73 modules in *E. coli*	This study
pNIC-FW	TGTGAGCGGATAACAATTCC	Confirmation	Addgene
pNIC-RV	AGCAGCCAACTCAGCTTCC	Confirmation	Addgene
cbpD-ddPCR-FW	ACCCGCTACTTCGACTTCTA	ddPCR	This study
cbpD-ddPCR-RV	CGCTGCCAGACGTTATAGAT	ddPCR	This study
lpmO$_{VA}$-ddPCR-FW	CGCGGCAAAATACCTGTTAC	ddPCR	This study
lpmO$_{VA}$-ddPCR-RV	CAACAGCTTGAACATGAGCC	ddPCR	This study
lpmO$_{EF}$-ddPCR-FW	CTCATGGTTACGTAGCAAGTCC	ddPCR	This study
lpmO$_{EF}$-ddPCR-RV	AAACCTGAGACACCTGCACT	ddPCR	This study

[*]The 5' extensions and enzyme sequences used for molecular cloning are printed in bold and underlined, respectively.

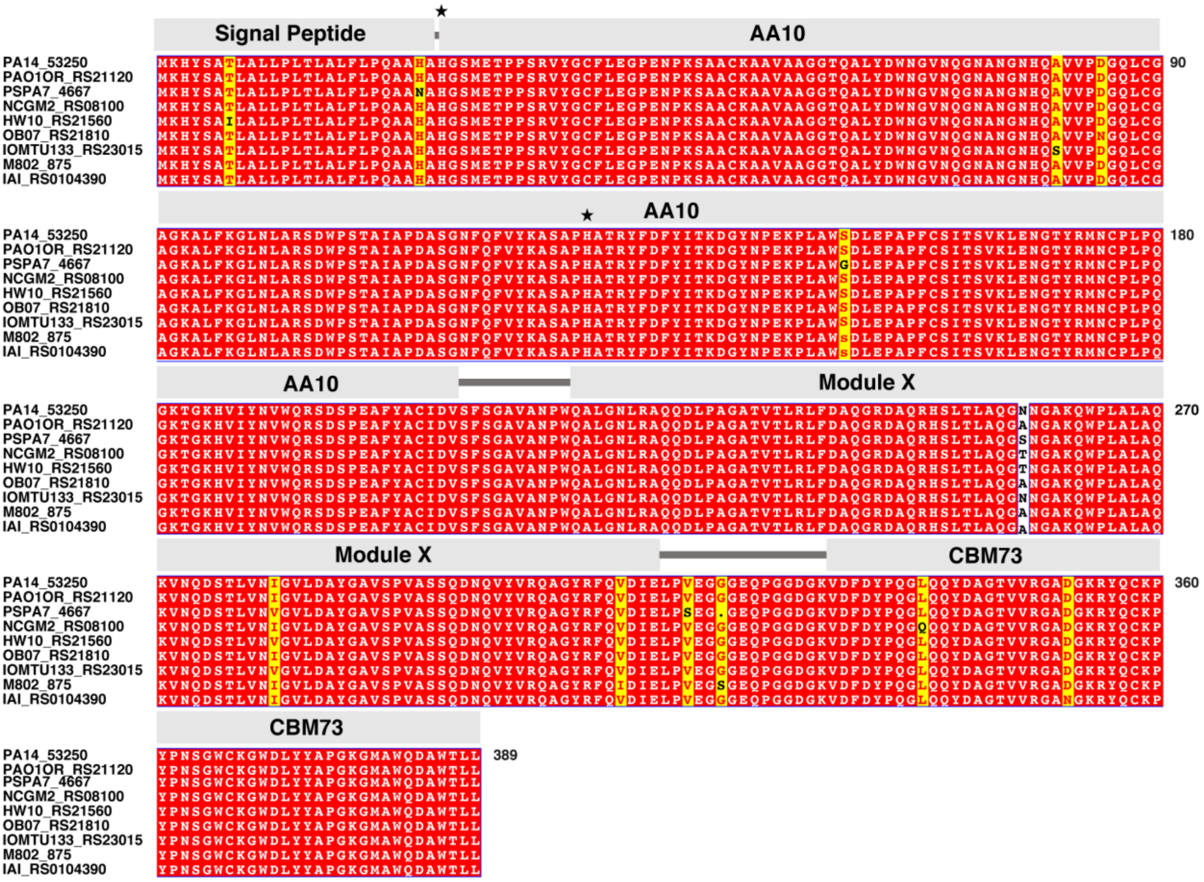

Supplementary Fig. 1. Multiple sequence alignment of CbpD sequences. Alignment of nine CbpD sequences originating from PA14 (WT), PAO1OR, PSPA7, NCGM2, HW10, OB07, IOMTU133, M802 and IAI, generated by MUSCLE [19] and formatted using ESPript 3.0 [20]. Identical and conserved residues are boxed in red. The black stars indicate the two histidine residues making up the histidine brace.

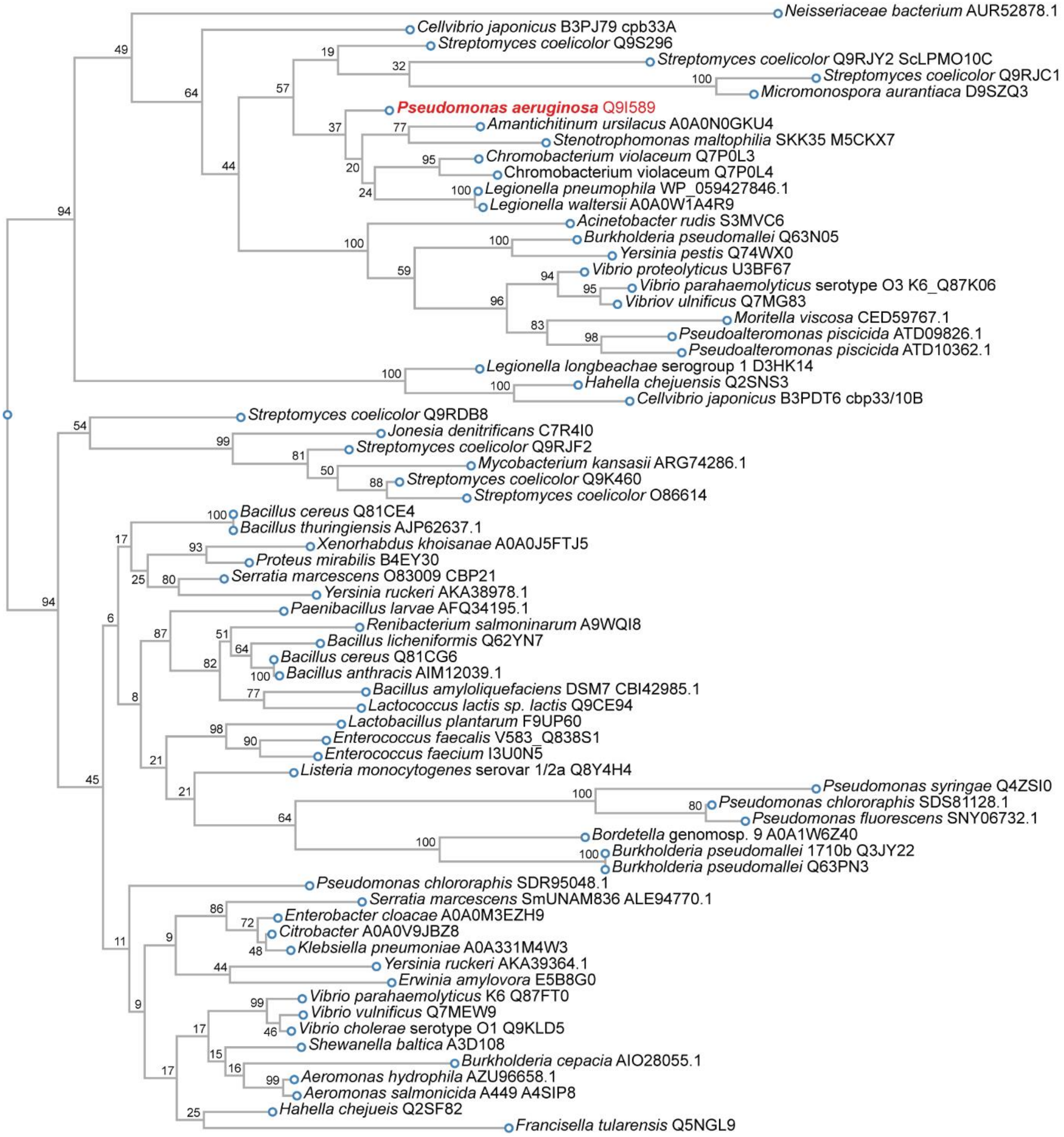

Supplementary Fig. 2. Phylogenetic tree of selected family AA10 LPMOs (AA10 modules only). The phylogenetic tree is based on a multiple sequence alignment of selected sequences made using MUSCLE (with default parameters) and was constructed using the "build" function of ETE3 v3.1.1 [21] that utilizes PhyML v20160115. Branch support values were computed from 100 bootstrapped trees. UniProt identifiers for the proteins are indicated next to the name of the bacterial species. CbpD is indicated in red-colored bold formatting.

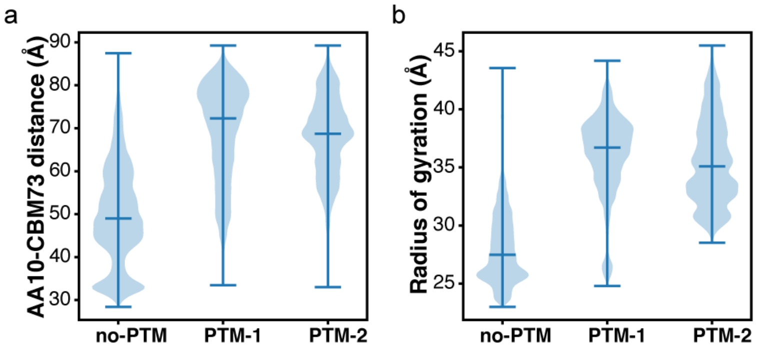

Supplementary Fig. 3. Effect of post-translational modifications on the predicted CbpD structure. Solution structures of CbpD were modeled based on data from 525 ns MD simulations of three models (no-PTM, PTM-1 and PTM-2), using 262,000 structures for each model. **a** For each structure in the MD trajectories, the distance between the center of mass of domains 1 (AA10) and 3 (CBM73) was measured. Distance distributions for the three models are shown as kernel density distributions in a violin plot. **b** Radius of gyration for each structure in the MD trajectories. Distributions of calculated radiuses are shown as kernel density distributions in violin plots. In both panels, the median, max and min values of the data sets are indicated by horizontal blue lines.

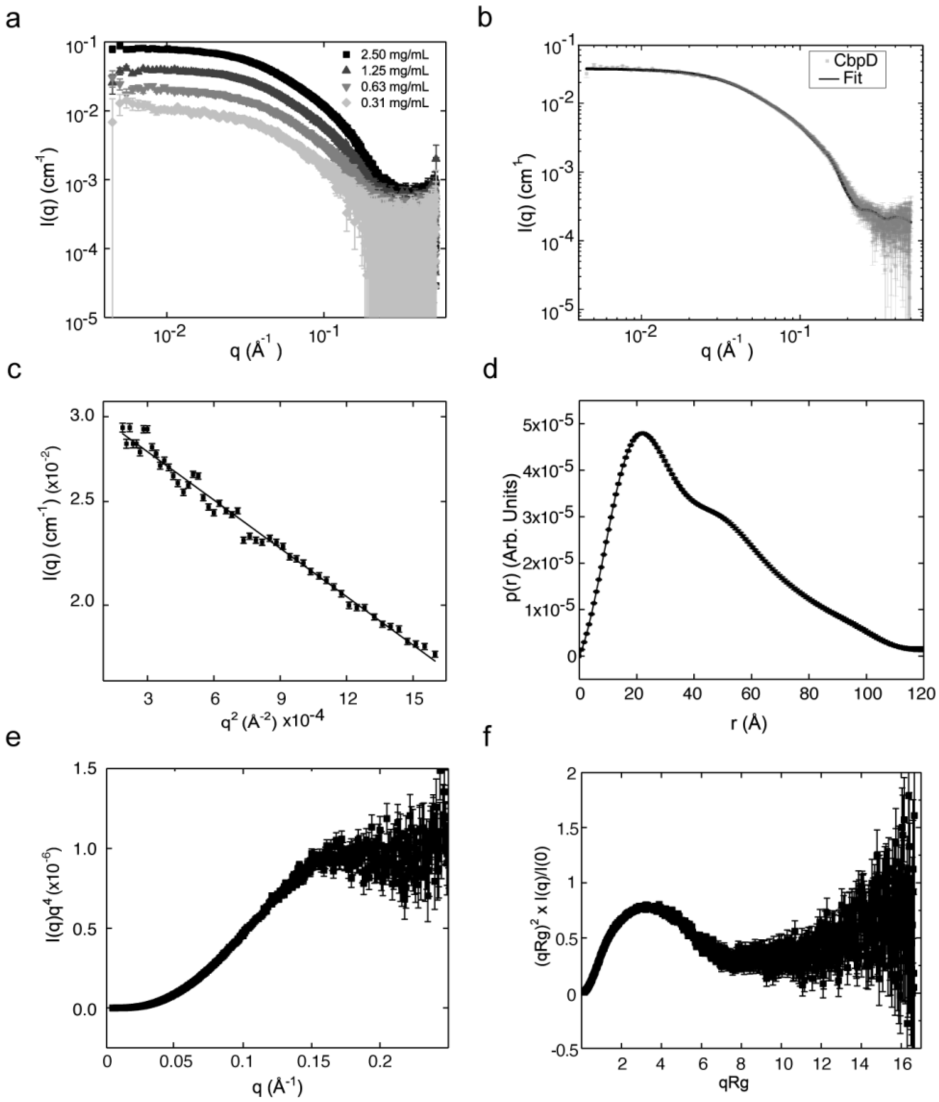

Supplementary Fig. 4. Small-Angle X-ray Scattering analysis of full-length rCbpD$_{EC}$. Synchrotron SAXS data were collected at ESRF BM29 [9] beamline. A data set extrapolated to zero solute concentration was generated for further analysis from four original data sets. **a** SAXS curves at 20 °C using multiple rCbpD$_{EC}$ concentrations. **b** Fit of atomistic *Pepsi-SAXS* [17] model to the CbpD SAXS data ($\chi 2$ = 2.35). **c** Guinier analysis. The linear fit in the Guinier region points to a monodisperse solution. The fit was also used to calculate *Rg*. **d** Pair-distance distribution function (*p(r)*). The asymmetric shape of the curve and the long decline from the maximum shows that CbpD is an elongated protein. CbpD has a maximum diameter of approximately 120 Å. **e** Porod plot, used for calculating the Porod volume. **f** Dimensionless Kratky plot. The bell-shaped plot suggests that the protein is folded, however, the maximum at $qRg \approx 3$ shows that it is not completely globular, but contains extended regions. A globular protein would have its maximum in the dimensionless Kratky plot around $qRg = \sqrt{3}$. Data are presented as mean ± SEM with respect to the number of pixels used in the data averaging. The experiment was performed once.

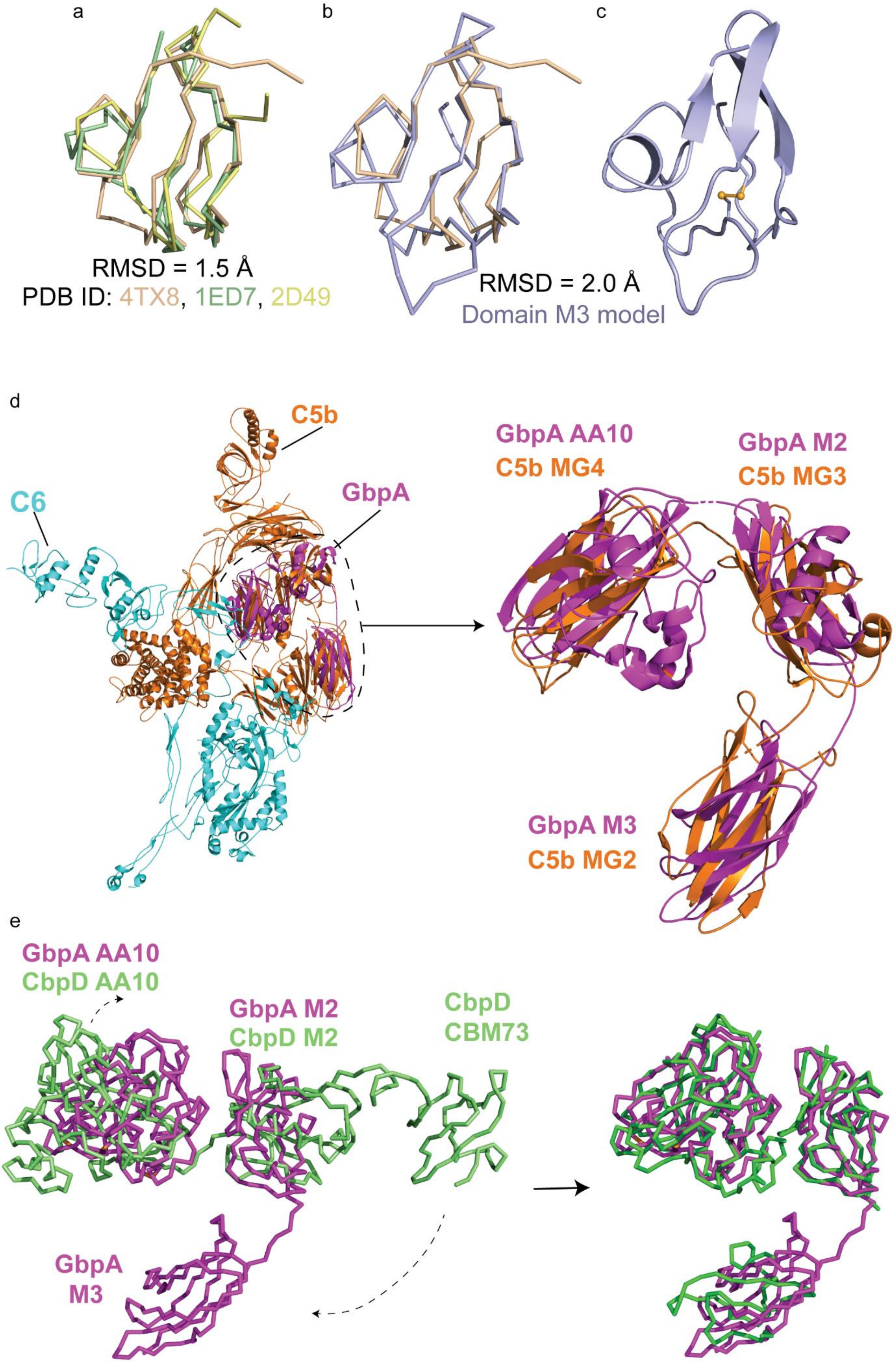
a
b
c
RMSD = 1.5 Å
PDB ID: 4TX8, 1ED7, 2D49
RMSD = 2.0 Å
Domain M3 model
d
C5b
C6
GbpA
GbpA AA10
C5b MG4
GbpA M2
C5b MG3
GbpA M3
C5b MG2
e
GbpA AA10
CbpD AA10
GbpA M2
CbpD M2
CbpD
CBM73
GbpA
M3

Supplementary Fig. 5. Homology modeling of CbpD domain 3 and structural similarity between GbpA and the complement factor C5-C6 complex. a Ribbon representation of superimposed CBM5 (PDB ID: 2D49) and CMB12 (PDB IDs: 4TX8 and 1ED7) domains that were used to predict the structure of the CBM73 domain of CbpD in RaptorX. **b** Snapshot of the MD-equilibrated third domain of CbpD (CBM73; violet), superimposed on the CBM12 domain of PDB ID: 4TX8 (beige). **c** Cartoon representation of CbpD domain 3 (CBM73). The predicted disulfide bridge is shown in ball and stick representation. **d** Structural similarity between GbpA and the C5-C6 complex identified by searching the DALI database [22] (Z-score = 10.2, RMSD = 13 Å from 1528 aligned atoms) The C5b-C6 complex (PDB ID: 4A5W) and GbpA (PDB ID: 2XWX) are shown in cartoon representation. The C5b and C6 molecules are colored orange and blue, respectively, and the GbpA molecule is colored purple. The right panel shows an enlarged image of GbpA superimposed on the MG2, -3 and -4 domains of C5b (see [23] for a detailed description of the C5b-C6 complex). **e** Superposition of GbpA (PDB ID: 2XWX; green colored ribbon) on the CbpD model fitted to the SAXS-data (purple colored ribbon), yielding a RMSD = 17 Å from 1364 aligned atoms. The right panel shows superpositioning of the individual CbpD modules on the GbpA structure, dashed arrows indicating the putative structural movement of the CbpD modules needed to obtain the GbpA 3D modular arrangement. The individual superpositions yielded RMSD values of 1.8 Å (705 atoms aligned), 1.9 Å (540 atoms aligned) and 8.0 Å (141 atoms aligned) for the AA10 modules M2 modules and M3/CBM73 modules, respectively. It should be noted that the GbpA M3 module has low structural similarity to the CbpD CMB73 model, but the overall shape is relatively similar, the latter being slightly smaller.

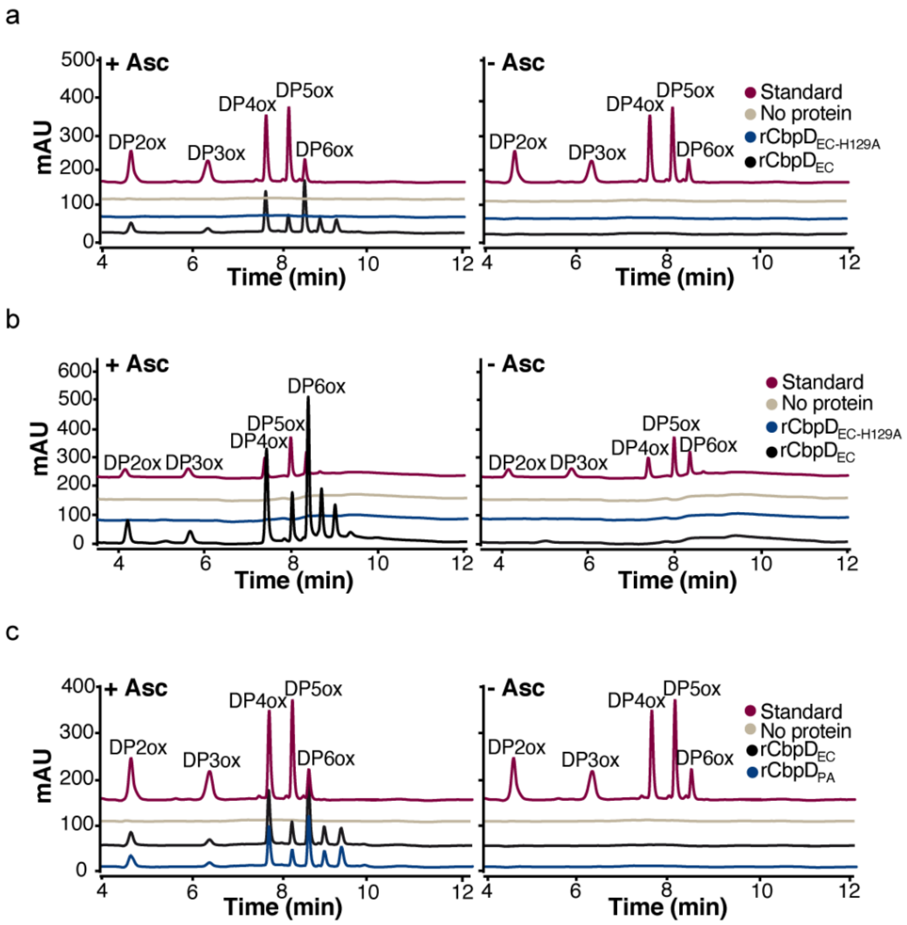

Supplementary Fig. 6. Enzymatic activity of CbpD variants toward β-chitin. The activity of 1µm rCbpD$_{EC}$ and rCbpD$_{EC-H129A}$ towards 10 mg·ml^{-1} β-chitin in 20 mM Tris-HCL pH 7.0 in the presence or absence of 1.0 mM ascorbate (Asc) was assessed by analyzing soluble products using hydrophilic interaction chromatography (HILIC). The chromatograms show products (= chitooligosaccharide aldonic acids) formed after 2h **a** and 24 h **b**. The degree of polymerization of the products is indicated by the *n* in DP*n*ox. **c** Comparison of rCbpD$_{EC}$ and rCbpD$_{PA}$ activity on ß-chitin. The reaction was set up as described in (**a**) and products were analyzed by HILIC after 2h incubation. Chromatograms are representative of three experiments.

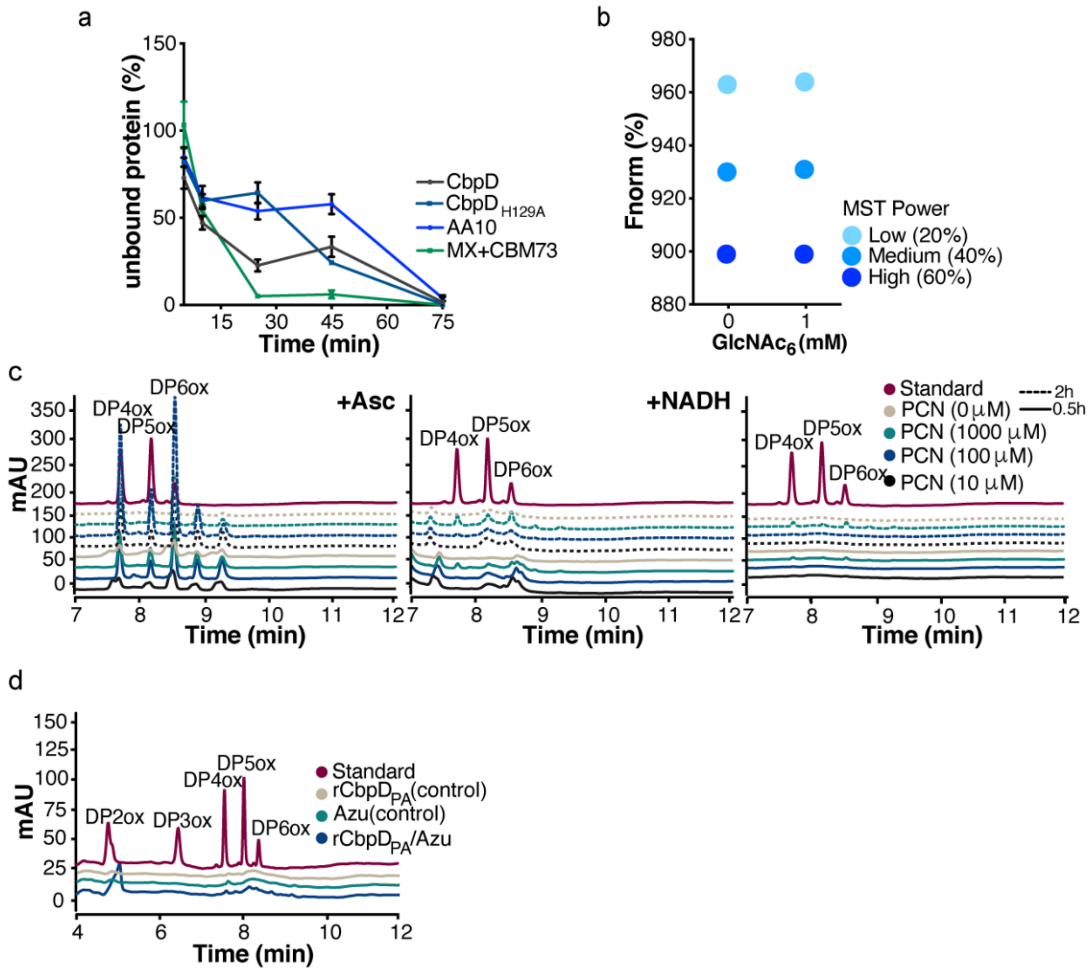

Supplementary Fig. 7. CbpD binding of β-chitin and chitohexaose, and effect of pyocyanin on CbpD activity. a Binding of 10 µM rCbpD$_{PA}$, rCbpD$_{PA-H129A}$, AA10 (AA10 module only) and MX+CBM73 (the MX and CBM73 domains only) to 10 mg·ml^{-1} β-chitin in 20 mM Tris-HCL pH 7.0, analyzed by quantification of the concentration of soluble protein over time, at 22 °C. The amount of unbound (soluble) protein in the presence of chitin is represented as the percentage of the amount unbound protein in the absence of chitin. The data are plotted as the mean ± SEM, representing three biological replicates. **b** Binding of 50 nM rCbpD to 1 mM hexa-*N*-acetyl-chitohexaose (GlcNAc$_6$, NAG6) evaluated by microscale thermophoresis (MST) analysis. The Y-axis indicates the change in fluorescence (normalized) at low (20%), medium (40%) or high (60%) MST power, for CbpD in the presence or absence of GlcNAc$_6$. **c** Effect of pyocyanin (PCN) on CbpD activity. Chromatograms (HILIC) show soluble reaction products emerging from the activity of 1µM rCbpD$_{EC}$ towards10 mg·ml^{-1} β-chitin in 20 mM Tris-HCL pH 7.0. The reactions were performed in the presence of 0, 10, 100 and 1000 µM pyocyanin and either 0.25 mM ascorbate (Asc, left panel) or 1.0 mM NADH (middle panel). At higher PCN concentrations (1000 µM), a decline in activity was observed most likely due to CbpD inactivation by excess H_2O_2, a trait also observed for other LPMOs. In the control reactions, no reducing agent (AA or NADH) was added (right panel). **d** Reaction products emerging in a reaction of 1 µM of the apo form of full-length rCbpD$_{PA}$ with 10 mg·ml^{-1} β-chitin in 20 mM Tris-HCL pH 7.0 analyzed by HILIC. All reactions contained Pyocyanin (100 µM), but no ascorbate. Azurin (Azu) was included in equimolar concentration to CbpD (1 µM) when indicated. Reactions were analyzed after 2h incubation at 37 °C. The chromatogram is representative of three experiments. Source data are provided as a Source Data file (a).

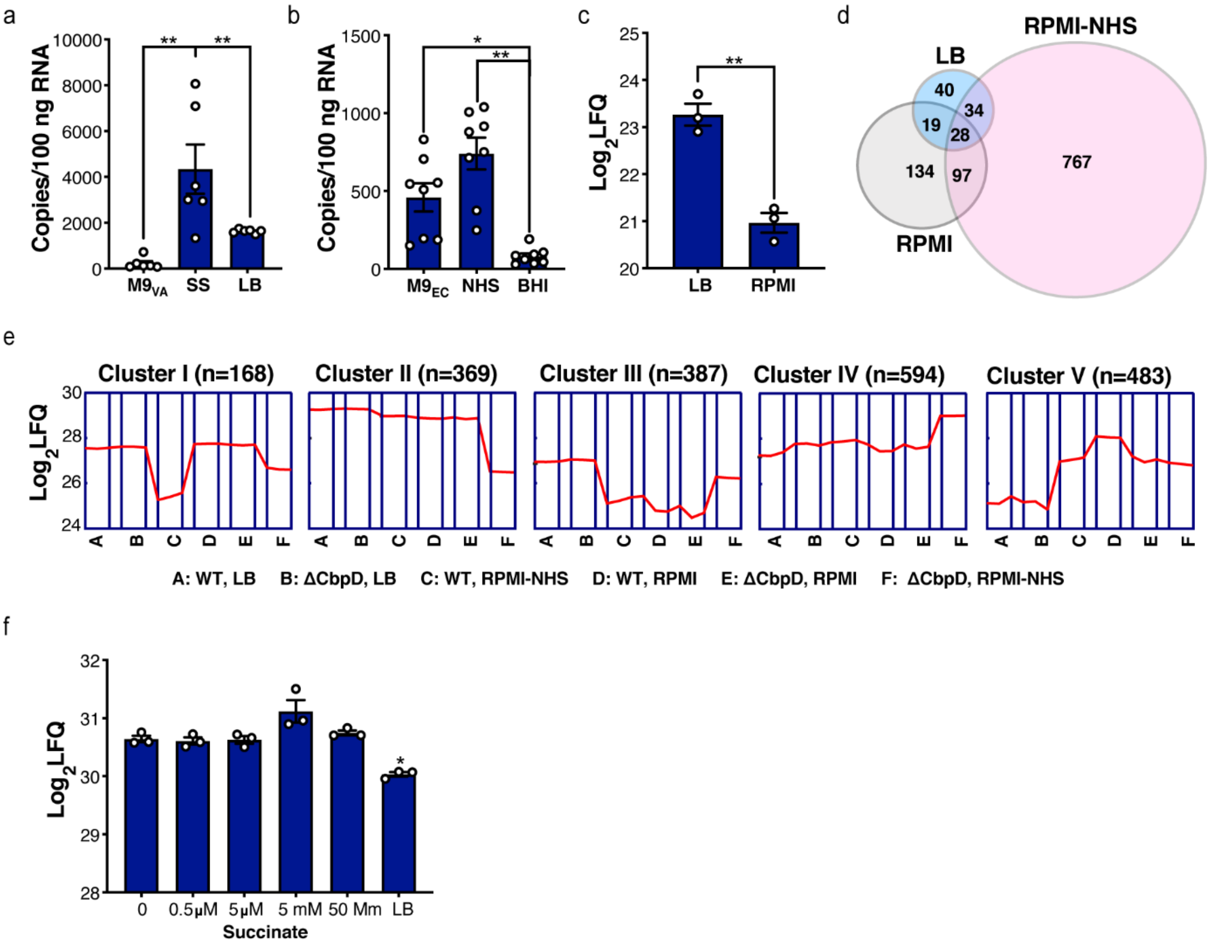

Supplementary Fig. 8. Quantification of *lpmo* expression and changes in the PA proteome under different growth conditions. Absolute quantification of mRNA transcripts of **a** *Vibrio anguilarum* ($lpmO_{VA}$) and **b** *Enterococcus faecalis* ($lpmO_{EF}$) grown in bacteriologic medium (LB or BHI) or in M9 minimal medium supplemented with 0 or 10% SS or NHS (30 min incubation), as indicated. The data are plotted as the mean ± SEM, representing three experiments performed in duplicate (*Vibrio anguilarum* ; VA) or triplicate (*Enterococcus faecalis;* EC) and analyzed by two-way ANOVA (Tukey's multiple comparisons test). (a) $M9_{VA}$ vs SS *p*=0.0024, SS vs LB *p*=0.0297; (b)*:* $M9_{EC}$ vs BHI *p*= 0.0166, NHS vs BHI 0.0002). NHS: Normal human serum, SS: Normal salmon serum, BHI: Brain heart infusion. **c** Secretion of CbpD by WT was assessed in the mid-exponential growth phase (OD_{600}=0.6) in LB and RPMI media using label-free quantitative (LFQ) proteomics. The data are plotted as the mean (log_2 LFQ) ± SEM, representing three biological replicates and analyzed by two-tailed *t* test (*p*=0.0018). **d** Venn diagram illustrating numbers of common and unique proteins, which the regulation was significantly differs between the wild-type and in the ΔCbpD deletion mutant during growth in different conditions (LB, RPMI or RPMI/NHS). **e** Trend plots showing average abundance profiles for each cluster of proteins, as identified by hierarchical clustering (**Fig. 2f**) of proteins expressed by WT and ΔCbpD in three different growth conditions (LB, RPMI and RPMI-NHS). The data on Y-axis presented as log_2 LFQ (Label-free quantification). **f** The effect of succinate on CbpD expression levels was assessed using quantitative proteomics**.** WT PA was grown in M9 (supplemented with glucose, $MgSO_4$, and $CaCl_2$) until reaching OD_{600}=0.4. The samples were supplemented with increasing concentrations of succinate (0, 0.5 μM, 5 μM, 5 mM and 50 mM) and harvested at OD_{600}=0.7-0.8. Bacteria grown in LB medium were included as controls. The data are plotted as the mean log_2 LFQ) ± SEM, representing three biological replicates and analyzed by two-way ANOVA (Tukey's multiple comparisons, absence of succinate vs LB, p=0.0129). When applicable the significance is indicated by asterisks (*): The significance is indicated by asterisks (*): **p* ≤ *0.05;* ***p* ≤ 0.01. Source data are provided as a Source Data file (a and b).

a b

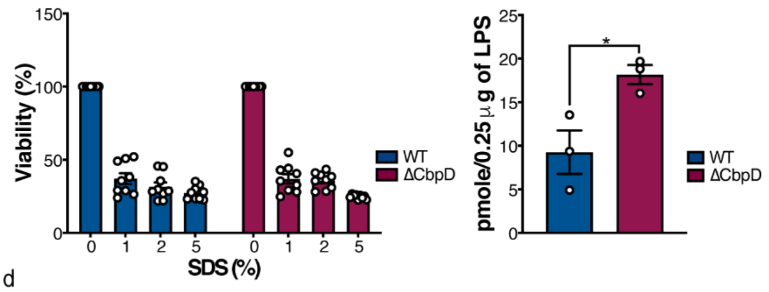

c

Mole percentage of monosaccharides present in LPS

Mole (%)	WT	ΔCbpD
Rha	31,51	31,98
Glc	44,23	44,80
GlcNAc	17,71	17,71
GalNAc	4,30	3,76
Hep	2,25	1,74

d

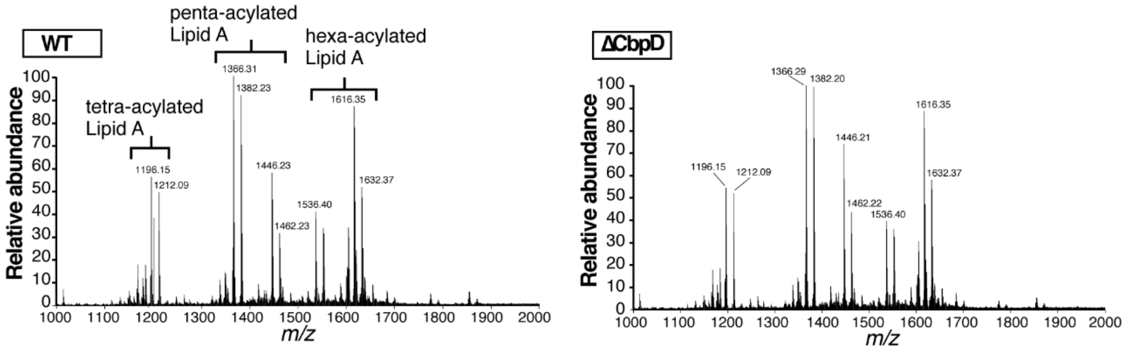

Supplementary Fig. 9. Effect of CbpD deletion on cell envelope stability, LPS quantification and lipid-A profile. a WT PA and the ΔCbpD strain grown at 37 °C in LB and lytic effect of SDS (0-5%) was measured as reduction in cell suspension turbidity (OD600) 5 minutes post-exposure. The OD600 of untreated bacterial cells (0% SDS) was arbitrarily set to 100% survival, and OD600 of treated bacterial cells is represented as percentage survival. The data are plotted as the mean ± SEM, representing three experiments performed in triplicate. **b** Quantification of Kdo in lipopolysaccharide (LPS) purified from WT and the ΔCbpD strain was determined by DMB-UPLC-FL. The data are plotted as the mean ± SEM, representing three biological replicates and analyzed by two-tailed *t* test (*p*=0.0320). The significance is indicated by asterisks (*): *$p \leq 0.05$. **c** Quantification of monosaccharides in LPS purified from WT and the ΔCbpD strain was determined by alditol acetate (AA) method. The data are shown as the mean of “mole percentage”, representing three biological replicates. **d** Representative ESI-MS analysis of lipid-A extracted from WT and the ΔCbpD strain grown at 37 °C in LB. Spectra were obtained in the negative ion mode, representing three biological replicates. The peaks representing the different Lipid A variants identified are indicated by their *m/z* values where 1196, 1366 and 1536 represent monophosphorylated tetra-, penta- and hexa-acylated lipid-A, respectively, where [M+16] indicates hydroxylation. Both penta- and hexa-acylated Lipid A variants are also present in their bisphosphorylated forms, represented by *m/z* peaks 1446 and 1616, respectively. These variants are also present in hydroxylated forms. Source data are provided as a Source Data file (a-c).

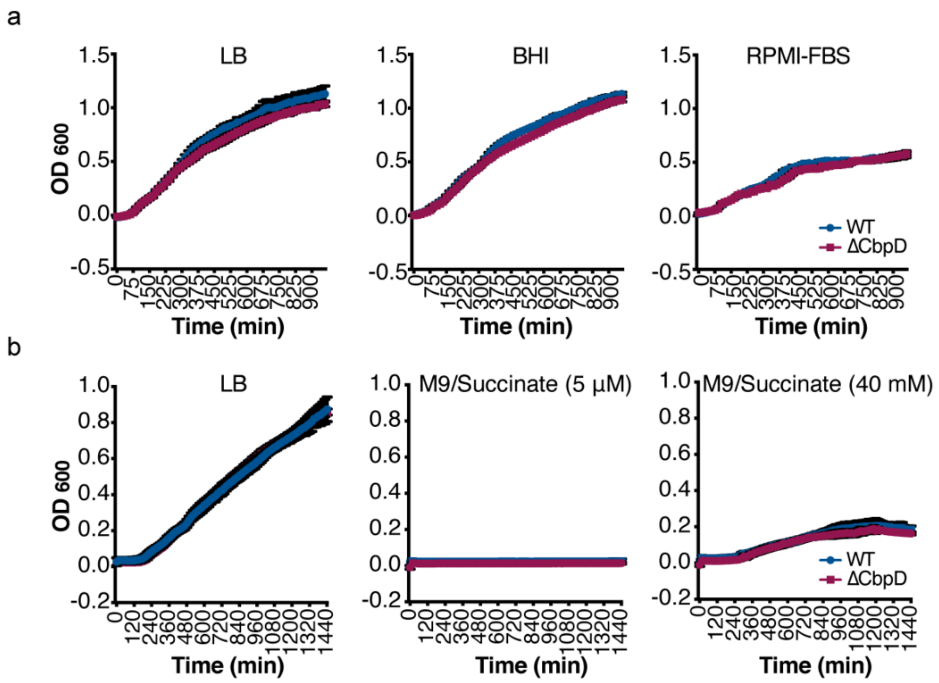

Supplementary Fig. 10. Growth of PA strains. Growth curves for WT (PA14) and the ΔCbpD strain grown at 37 °C in **a** LB, BHI or RPMI-FBS (10%) (generated by Synergy H1 Hybrid Reader) and **b** M9 (containing $MgSO_4$, $CaCl_2$) supplemented with succinate (5 µM and 40 mM) as sole carbon source. The LB medium is included as an extra control (generated by Varioskan™ LUX). The data are plotted as the mean ± SD, representing two experiments performed in duplicate (panel a) or triplicate (panel b).

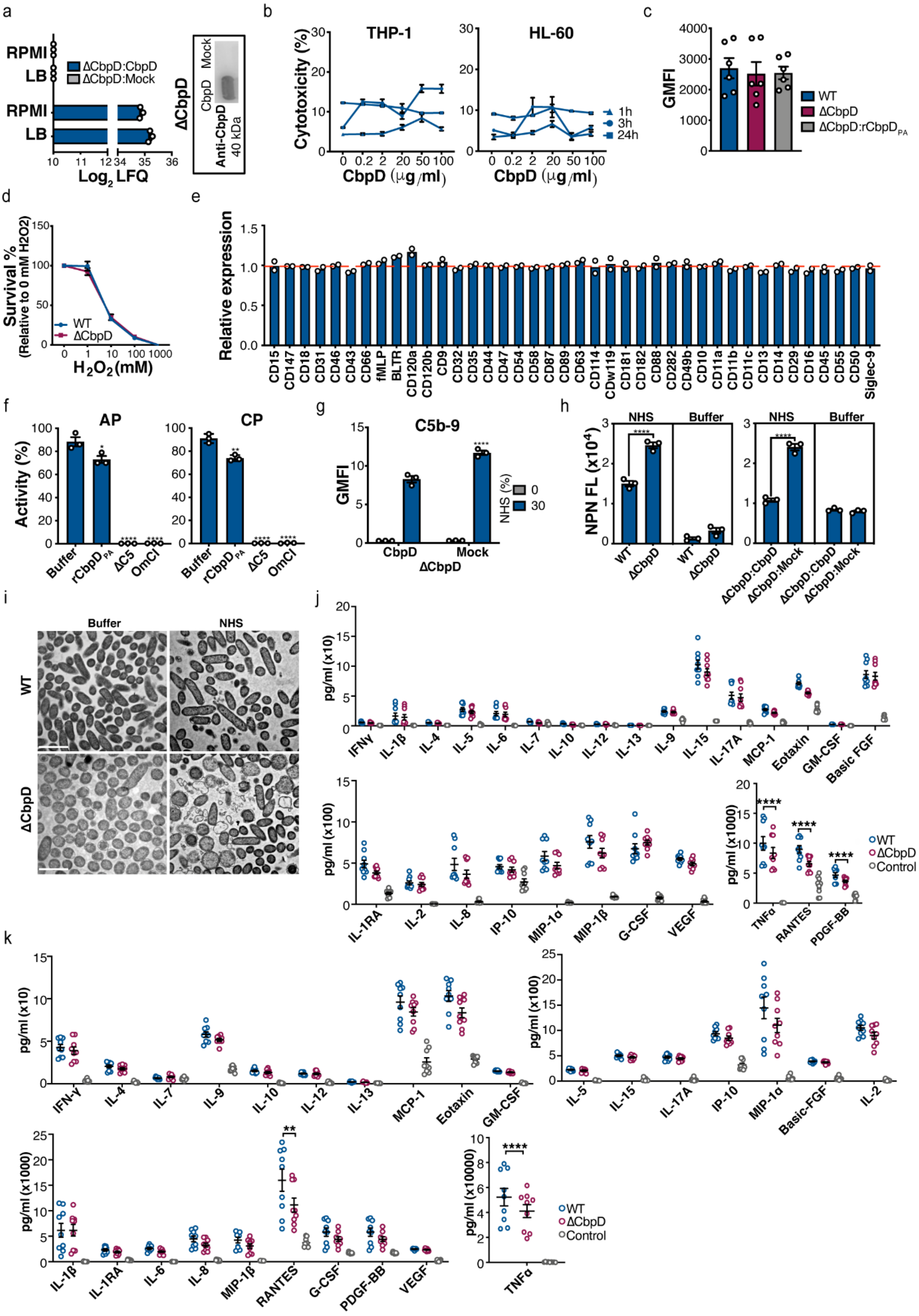
a
RPMI
LB
ΔCbpD:CbpD
ΔCbpD:Mock
Log2 LFQ
ΔCbpD
CbpD
Mock
Anti-CbpD
40 kDa
b
THP-1
HL-60
Cytotoxicity (%)
CbpD (μg/ml)
1h
3h
24h
c
GMFI
WT
ΔCbpD
ΔCbpD:rCbpDPA
d
Survival %
(Relative to 0 mM H2O2)
H2O2(mM)
WT
ΔCbpD
e
Relative expression
CD15
CD147
CD18
CD31
CD46
CD43
CD66
fMLP
BLTR
CD120a
CD120b
CD9
CD32
CD35
CD44
CD47
CD54
CD58
CD87
CD89
CD63
CD114
CDw119
CD181
CD182
CD88
CD282
CD49b
CD10
CD11a
CD11b
CD11c
CD13
CD14
CD29
CD16
CD45
CD55
CD50
Siglec-9
f
AP
CP
Activity (%)
Buffer
rCbpDPA
ΔC5
OmCI
g
C5b-9
GMFI
NHS (%)
CbpD
Mock
ΔCbpD
h
NHS
Buffer
NPN FL (x10^4)
WT
ΔCbpD
ΔCbpD:CbpD
ΔCbpD:Mock
i
Buffer
NHS
WT
ΔCbpD
j
pg/ml (x10)
IFNγ
IL-1β
IL-4
IL-5
IL-6
IL-7
IL-10
IL-12
IL-13
IL-9
IL-15
IL-17A
MCP-1
Eotaxin
GM-CSF
Basic FGF
pg/ml (x100)
IL-1RA
IL-2
IL-8
IP-10
MIP-1α
MIP-1β
G-CSF
VEGF
pg/ml (x1000)
TNFα
RANTES
PDGF-BB
WT
ΔCbpD
Control
k
pg/ml (x10)
IFN-γ
IL-4
IL-7
IL-9
IL-10
IL-12
IL-13
MCP-1
Eotaxin
GM-CSF
pg/ml (x100)
IL-5
IL-15
IL-17A
IP-10
MIP-1α
Basic-FGF
IL-2
pg/ml (x1000)
IL-1β
IL-1RA
IL-6
IL-8
MIP-1β
RANTES
G-CSF
PDGF-BB
VEGF
pg/ml (x10000)
TNFα
WT
ΔCbpD
Control

Supplementary Fig. S11. *Ex vivo* and *in vitro* analysis of CbpD virulence properties. a CbpD expression in *trans* complemented ΔCbpD construct (ΔCbpD:CbpD) proteome and secretome analyzed by LC–MS/MS and immunoblotting, respectively. Bacteria were grown to exponential phase in LB medium or RPMI/HSA containing 10% LB. Media were supplemented with carbenicillin and arabinose. (ΔCbpD:Mock) served as negative controls. The data are plotted as the mean ± SEM, representing three biological replicates. The uncropped western blot is shown in supplementary Fig. S15. **b** Cytotoxicity of rCbpD$_{PA}$ toward immune cells (THP-1 and differentiated HL-60 cell lines), monitored by measuring LDH release. The data is expressed as a percentage of cytotoxicity and plotted as the mean ± SEM, representing three experiments performed in duplicate. **c** Whole blood phagocytosis of FITC-labeled WT and ΔCbpD. The data are plotted as the GMFI ± SEM, representing three experiments performed in duplicate. The gating strategy is shown in Supplementary Figure 17a. **d** WT PA and ΔCbpD survival following treatment with H_2O_2. The rate of survival of untreated bacterial cells were arbitrarily set to 100%, and the survival of treated bacterial cells is represented as percentage survival. The data are plotted as the mean ± SEM, representing three experiments performed in duplicate. **e** Competition between CbpD and receptor-specific monoclonal antibodies (mAbs) for binding to surface-expressed receptors on neutrophils. The amount of antibody binding to untreated cells was arbitrarily set equal to 1, and the ratio of antibody binding to CbpD-treated cells is represented as relative expression. The data are plotted as the geometric mean, representing two biological replicates. The gating strategy is shown in Supplementary Figure 17. **f** Activation of the classical and alternative complement pathways (labeled AP and CP, respectively) by rCbpD$_{PA}$ (20 µg·ml^{-1}) using the WIELISA assay. OmCI (20 µg·ml^{-1}) and C5 depleted NHS (ΔC5) were included as controls. The data are plotted as the mean ± SD, representing three biological replicates and analyzed by two-way ANOVA (Tukey's multiple comparisons). AP: buffer vs rCbpD$_{PA}$ *p*=0.0218, buffer vs ΔC5 p= 1.592759191E-6, buffer vs OMCI *p*=1.602145941E-6; CP: buffer vs rCbpD$_{PA}$ *p*=0.0011, buffer vs ΔC5 p=8.3011242E-8, buffer vs OMCI *p*=8.3570030E-8. **g** Binding of C5b-9 to *in trans* complemented ΔCbpD (ΔCbpD:CbpD/ ΔCbpD:Mock) surface evaluated by flow cytometry. C5b-9 deposition was identified using Alexa 488-labeled C5b-9. The data are plotted as the GMFI± SEM, representing three biological replicates. The gating strategy is provided in Supplementary Figure 16. The data were analyzed by two-way ANOVA (Sidak's multiple comparisons, NHS (30%) *p*= 2.5495771678E-5). **h** N-phenylnaphthylamine (NPN) fluorescent assay measuring outer membrane permeability of WT and ΔCbpD and *in trans* complemented ΔCbpD constructs (ΔCbpD:CbpD/ ΔCbpD:Mock) in either RPMI/HSA (buffer) or RPMI/HSA containing 10% pooled NHS. The data are plotted as the florescence intensity ± SEM, representing three biological replicates and analyzed by two-way ANOVA (Sidak's multiple comparisons). NHS: WT vs ΔCbpD *p*=1.0191110412E-5, ΔCbpD:CbpD vs ΔCbpD:Mock *p*= 1.10095958E-7. **i** Representative TEM electron micrographs of WT and ΔCbpD in the absence or presence of NHS (1h, 10%). The scale bar is 2 µm. The experiment was performed twice. **j and k** Cytokine profiling of whole human blood (associated with Figure 3g). Plasma harvested from blood samples infected with WT or ΔCbpD at 1h (j) and 3h (k) post -infection. The data are plotted as the mean ± SEM, representing three experiments performed in triplicate. The significant difference between WT and ΔCbpD is indicated by asterisks (*). Data were analyzed by two-way ANOVA (Tukey's multiple comparisons) and the significant difference between WT and ΔCbpD is indicated by asterisks. Left panel (1h): TNF *p*= 5.2816212E-8, RANTES *p*<1E-15, PDGF-BB *p*=0.0007; Left panel (3h): TNF *p*<1E-15, RANTES *p*=0.0029. The statistical significance is indicated by asterisks (*): **p* ≤ *0.05;* ***p* ≤ 0.01; ****p* ≤ 0.001; *****p* ≤ 0.0001. Source data are provided as a Source Data file (b-h and j-k).

a

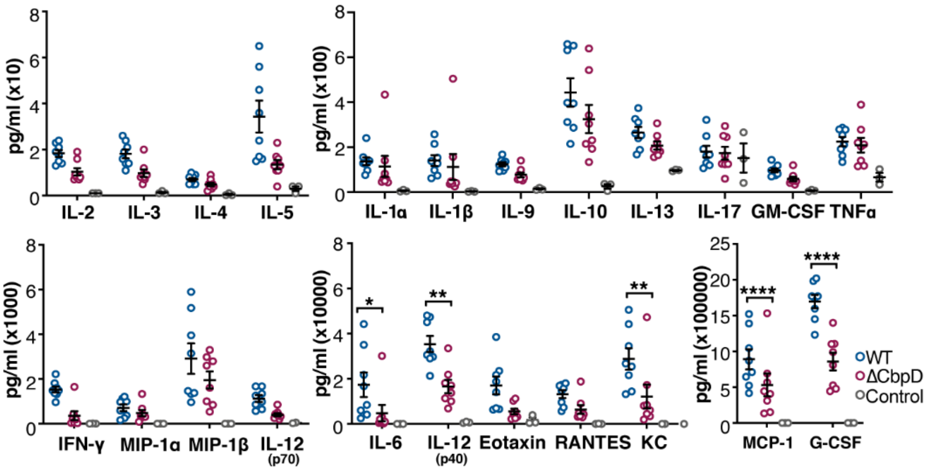

b

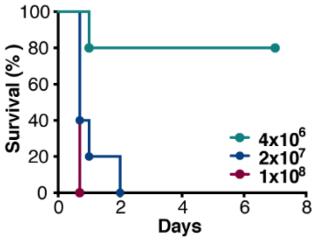

Supplementary Fig. 12. *In vivo* analysis of CbpD virulence. a Concentration of cytokines, chemokines or growth factors in the serum of CD-1 mice 4h post infection with WT and ΔCbpD (associated with Fig. 4c). The data are plotted as the mean ± SEM, representing n=16 biologically independent animals (8 WT and 8 ΔCbpD infected mice). Mock-infected mice (n=3 biologically independent animals) were included as control. The data were analyzed by two-way ANOVA (Dunnett's multiple comparisons test) and the significant difference between WT and ΔCbpD is indicated by asterisks (*): *$p \leq 0.05$; **$p \leq 0.01$; ***$p \leq 0.001$; ****$p \leq 0.0001$. IL-6: p=0.0497, IL-12 (p40): p=0.0018, G-CSF p<0.0001, KC: p=0.0063, MCP-1: p<0.0001. **b** CD1 mice were intravenously infected with different amounts of WT (CFU/mice) to determine the optimal dosage for survival experiments. Data represent n=10 biologically independent animalsa (5 WT and 5 ΔCbpD infected mice). Survival is represented by Kaplan-Meier survival curves. Source data are provided as a Source Data file (a-b).

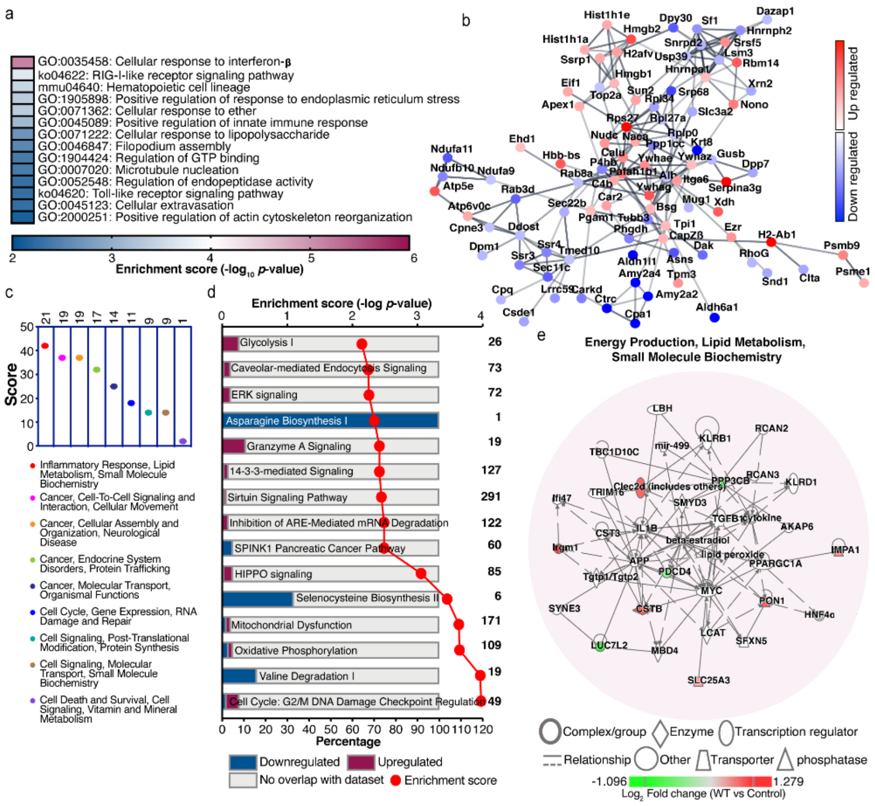

Supplementary Fig. 13. The effect of CbpD on the splenic proteome during systemic infection *in vivo.* a Pathways enriched in the shared splenic proteome of the WT-/ΔCbpD-infected vs uninfected mice. The enrichment score (-log10 *p*-value, cut off=0.01) was calculated by Metascape from which the *p* value was calculated based on the cumulative hypergeometric distribution. **b** STRING network analysis showing the connection of regulated proteins in the unique spleen proteome of ΔCbpD-infected mice vs control (Supplementary Data 10). The average fold change values of up- or down-regulated proteins are mapped to the nodes and is visualized using a blue-white-red gradient. Proteins without any interaction partners within the network (singletons) are omitted from the visualization**. c** The score of the molecular networks associated with regulated proteins (Supplementary Data 10) in the unique spleen proteome of ΔCbpD-infected vs control mice. In total, 119 out of 128 uniquely regulated proteins (Supplementary Data 10) were mapped to the database, and nine networks were identified by Ingenuity Pathway Analysis (IPA) (see Supplementary Fig. 14 for details). The number of the focus molecules (differentially regulated) associated with each network is presented on top of the chart. **d** IPA canonical pathways significantly altered in ΔCbpD-infected mice. The bottom X-axis shows the percentage of upregulated (red), downregulated (blue), and proteins not overlapping with other data set (white) in each pathway. The top X-axis shows the enrichment score (-log *p*-value) for each pathway (cut off=0.01) as indicated by the red dots. The *p* value was calculated by right-tailed fisher's exact test. The numbers to the right of each bar show the total number of proteins in that particular canonical pathway. **e** Molecular and disease-based protein network detected by IPA analysis. The network is associated with the regulated proteins in the unique spleen proteome of WT-infected (Supplementary Data 10) vs control mice. The average fold change value of up- or down-regulated proteins are mapped to the nodes and are visualized using a green-white-red gradient. The shapes represent the molecular classes of the proteins (see legend to supplementary Fig. 14). Direct and indirect connections are presented with solid and dashed arrows, respectively.

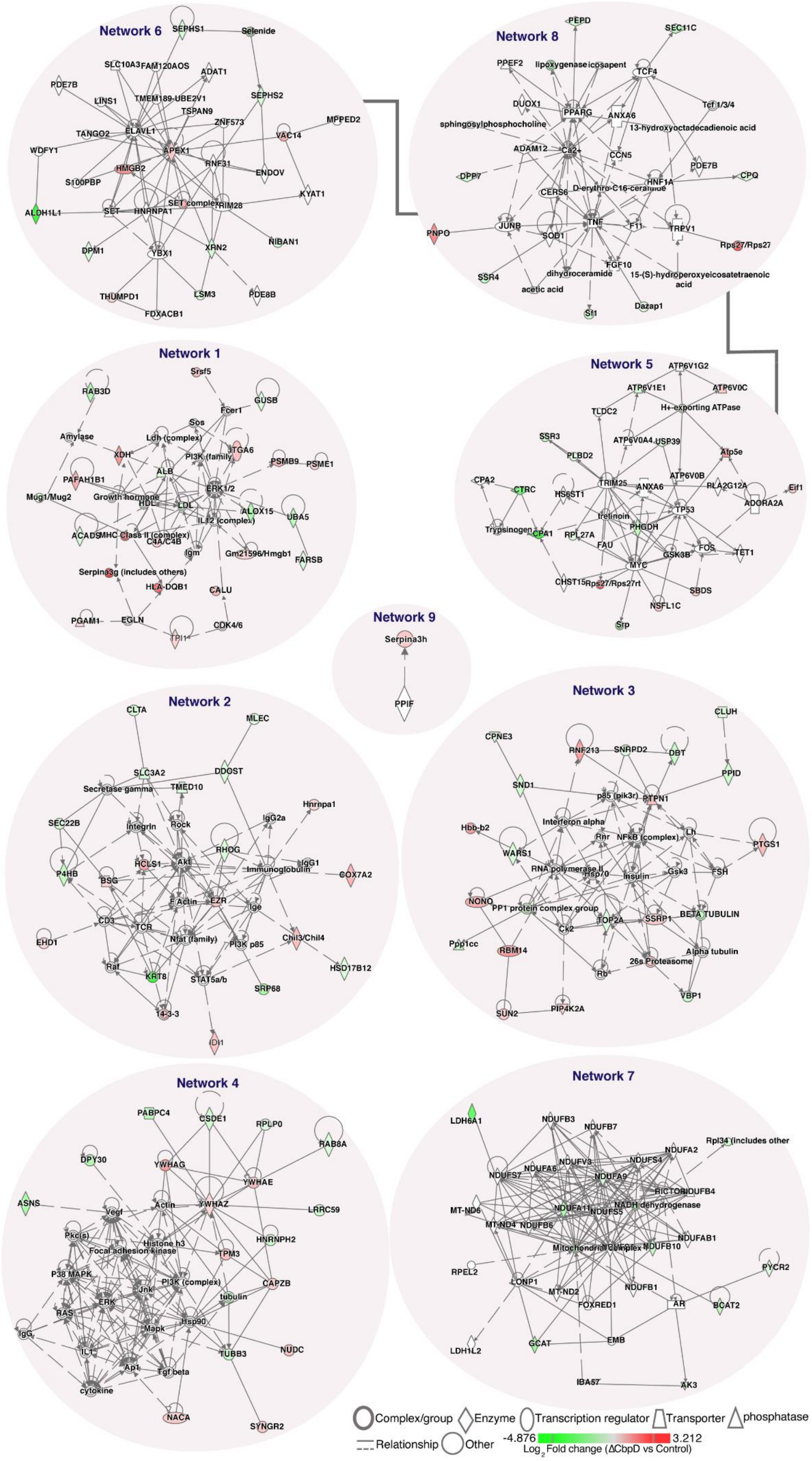
Network 6
SEPHS1
Selenide
SLC10A3
FAM120AOS
ADAT1
PDE7B
LINS1
TMEM189-UBE2V1
SEPHS2
TSPAN9
MPPED2
ZNF573
TANGO2
ELAVL1
VAC14
WDFY1
APEX1
HMGB2
RNF31
ENDOV
S100PBP
KYAT1
SET complex
TRIM28
ALDH1L1
SET
HNRNPA1
NIBAN1
DPM1
YBX1
XRN2
THUMPD1
LSM3
PDE8B
FDXACB1
Network 8
PEPD
SEC11C
PPEF2
lipoxygenase
icosapent
TCF4
DUOX1
PPARG
Tcf 1/3/4
ANXA6
sphingosylphosphocholine
13-hydroxyoctadecadienoic acid
ADAM12
Ca2+
CCN5
PDE7B
DPP7
HNF1A
CPQ
CERS6
D-erythro-C16-ceramide
PNPO
JUNB
TNF
F11
TRPV1
SOD1
Rps27/Rps27
FGF10
SSR4
dihydroceramide
15-(S)-hydroperoxyeicosatetraenoic acid
acetic acid
Dazap1
Sf1
Network 1
Srsf5
RAB3D
GUSB
Fcer1
Sos
Amylase
Ldh (complex)
ITGA6
XDH
PI3K (family)
PSMB9
PSME1
ALB
PAFAH1B1
ERK1/2
Mug1/Mug2
Growth hormone
HDL
LDL
ALOX15
UBA5
IL12 (complex)
ACADS
MHC Class II (complex)
C4A/C4B
Igm
Gm21596/Hmgb1
FARSB
Serpina3g (includes others)
HLA-DQB1
CALU
PGAM1
EGLN
TPI1
CDK4/6
Network 5
ATP6V1G2
ATP6V1E1
ATP6V0C
H+-exporting ATPase
TLDC2
SSR3
ATP6V0A4
USP39
PLBD2
Atp5e
ATP6V0B
CPA2
TRIM25
CTRC
HS6ST1
ANXA6
PLA2G12A
Eif1
ADORA2A
TP53
tretinoin
PHGDH
Trypsinogen
CPA1
RPL27A
FAU
GSK3B
FOS
TET1
MYC
CHST15
Rps27/Rps27rt
SBDS
NSFL1C
Srp
Network 9
Serpina3h
PPIF
Network 2
CLTA
MLEC
SLC3A2
DDOST
Secretase gamma
TMED10
Hnrnpa1
SEC22B
Integrin
Rock
IgG2a
RHOG
P4HB
HCLS1
Akt
IgG1
BSG
Immunoglobulin
COX7A2
F Actin
EZR
Ige
CD3
TCR
Nfat (family)
PI3K p85
Chil3/Chil4
EHD1
HSD17B12
Raf
KRT8
STAT5a/b
SRP68
14-3-3
IDI1
Network 3
CLUH
CPNE3
RNF213
SNRPD2
DBT
PPID
SND1
p85 (pik3r)
PTPN1
Hbb-b2
Interferon alpha
Lh
Rnr
NFkB (complex)
PTGS1
WARS1
RNA polymerase II
Hsp70
Insulin
Gsk3
FSH
NONO
PP1 protein complex group
TOP2A
SSRP1
BETA TUBULIN
Ppp1cc
Ck2
RBM14
Alpha tubulin
26s Proteasome
Rb
VBP1
SUN2
PIP4K2A
Network 4
PABPC4
CSDE1
RPLP0
RAB8A
DPY30
YWHAG
YWHAE
ASNS
Actin
YWHAZ
LRRC59
Vegf
Pkc(s)
Histone h3
Focal adhesion kinase
TPM3
HNRNPH2
P38 MAPK
PI3K (complex)
CAPZB
tubulin
ERK
Jnk
RAS
Hsp90
Mapk
IgG
IL1
TUBB3
NUDC
Ap1
Tgf beta
cytokine
NACA
SYNGR2
Network 7
LDH6A1
NDUFB3
NDUFB7
Rpl34 (includes other
NDUFA2
NDUFS4
NDUFA6
NDUFV3
NDUFS7
NDUFA9
RICTOR
NDUFB4
MT-ND6
NADH dehydrogenase
NDUFA11
NDUFS5
MT-ND4
NDUFB6
NDUFAB1
Mitochondrial complex
NDUFB10
RPEL2
PYCR2
LONP1
MT-ND2
NDUFB1
AR
FOXRED1
BCAT2
LDH1L2
GCAT
EMB
IBA57
AK3
Complex/group
Enzyme
Transcription regulator
Transporter
phosphatase
Relationship
Other
-4.876
3.212
Log2 Fold change (ΔCbpD vs Control)

Supplementary Fig. 14. Molecular and disease-based protein networks identified by IPA. Regulated proteins in the unique spleen proteome of ΔCbpD-infected mice vs control were used for IPA analysis (Supplementary Data 10). The score and rank of the networks are presented in supplementary Fig. 13c. Direct and indirect connections are presented with solid and dashed arrows, respectively. The average fold change value of up- or down-regulated proteins are mapped to the nodes and are visualized using a green-white-red gradient. The shapes represent the molecular classes of the proteins. Network 1: Inflammatory response, lipid metabolism, small molecule biochemistry; Network 2: cancer, cell-to-cell signaling and interaction, cellular movement; Network 3: cancer, cellular assembly and organization, neurological disease; Network 4: cancer, endocrine system disorders, protein trafficking, Network 5: cancer, molecular transport, organismal functions; Network 6: cell cycle, gene expression, RNA damage, and repair; Network 7: cell signaling, post-translational modification, protein synthesis; Network 8: cell signaling, molecular transport, small molecule biochemistry; Network 9: cell death and survival, cell signaling, vitamin, and mineral metabolism.

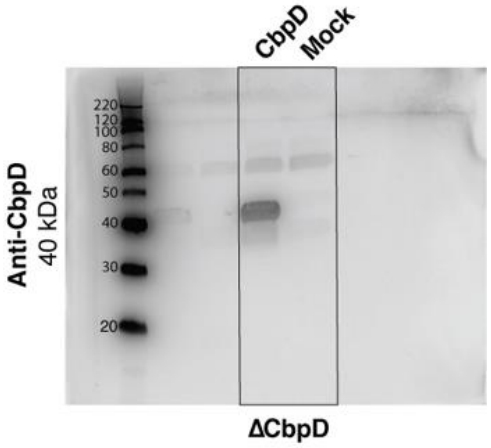

Supplementary Fig. 15. Identification of CbpD by immunoblotting. CbpD expression in *trans* complemented ΔCbpD construct (ΔCbpD:CbpD) secretome was analyzed by immunoblotting. Bacteria were grown to exponential phase in LB supplemented with carbenicillin and arabinose. The *in trans* complemented ΔCbpD with mock plasmid (ΔCbpD:Mock) served as negative controls. The Western blot shown represents the uncropped western corresponding to Supplementary Figure 11a**.**

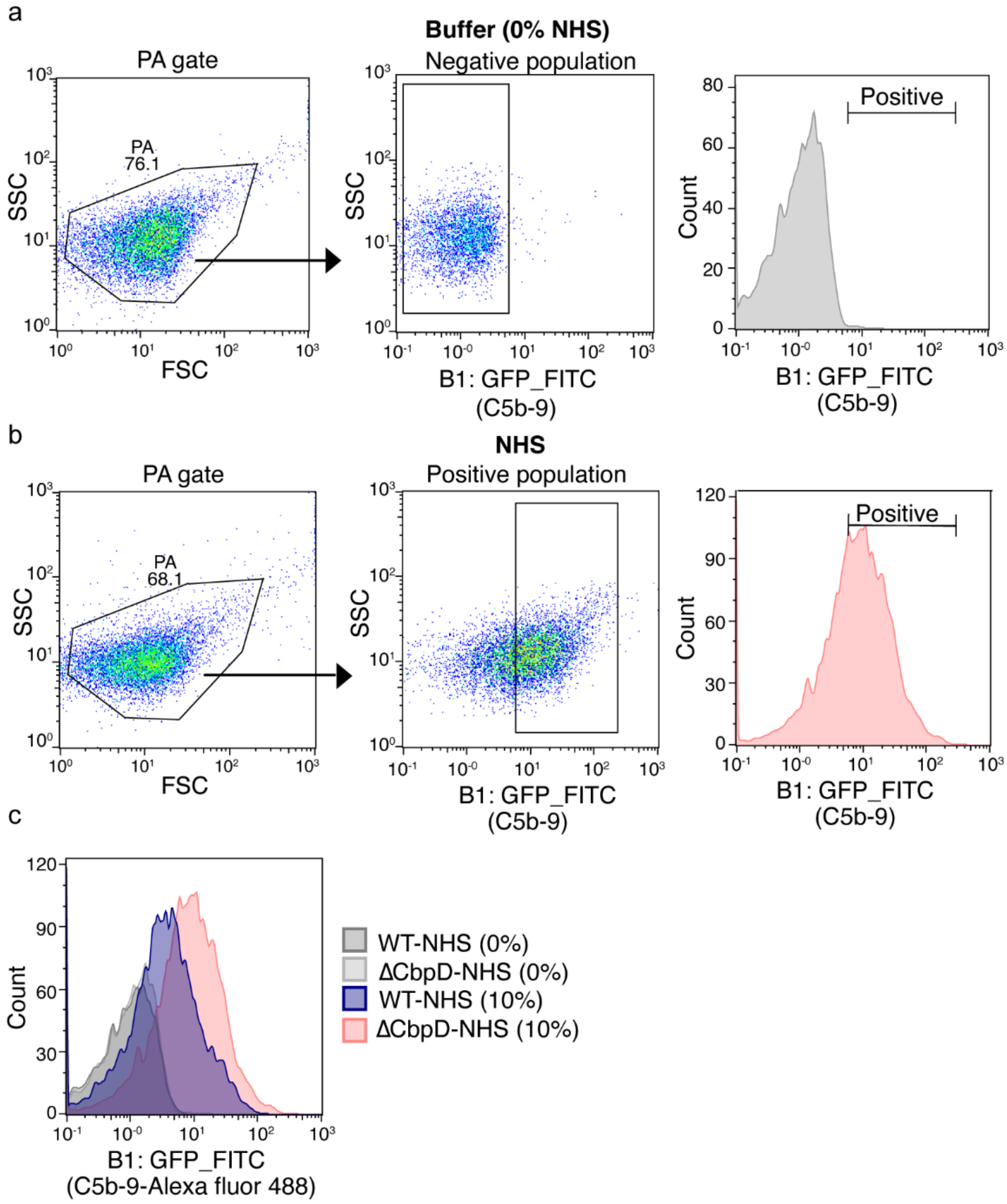

Supplementary Fig. 16. Gating strategy. The figure shows representative plots for gating strategy used for flow cytometric data presented in Fig. 3h-i and supplementary Fig. 11g. a, b The data were analysed in FlowJo and bacterial cells were gated on SSC versus FSC (left panel, PA gate). The geometric mean of the fluorescence intensity (GMFI) of the cells inside the gate was determined. The C3b^{+}, C5b-9^{+} and Bb^{+} cells in the NHS treated bacterial cells (b, middle and left panels) were identified based on the gating for the negative control (buffer) (a, middle and left panels). The percentage of the cells within bacterial gating is provided in the source file. **c** The representative histogram shows an overlay of WT and ΔCbpD incubated with buffer or NHS.

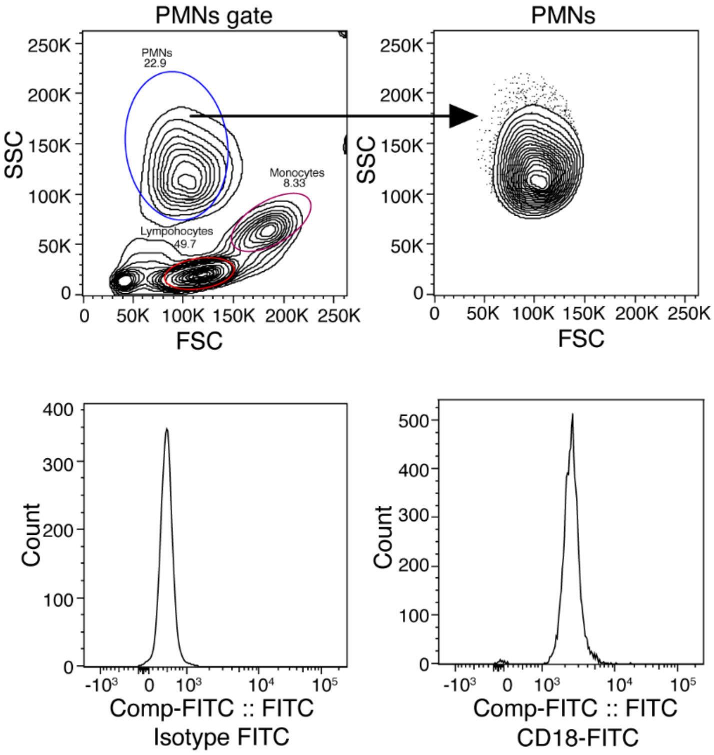

Supplementary Fig. 17. Gating strategy. The figure shows representative plots for gating strategy used for flow cytometric data presented in Fig. 11d and 11e. a The data were analysed in FlowJo and PMNs were gated on SSC versus FSC (left panel) based on size and granularity. The GMFI of the PMN cells inside the gate was determined. The percentage of the cells in the PMNs gate is provided in the source file or in the representative forward-sideward scatter plot. **b** The representative histogram associated with Figure 11e shows the isotype control and staining of PMNs with a fluorescent labeled antibody.

SI References

1. Lombard V, Ramulu HG, Drula E, Coutinho PM, Henrissat B. The carbohydrate-active enzymes database (CAZy) in 2013. *Nucleic Acids Res* **42**, 490-495 (2014).
2. Gaviard C, Cosette P, Jouenne T, Hardouin J. LasB and CbpD virulence factors of *Pseudomonas aeruginosa* carry multiple post-translational modifications on their lysine residues. *J Proteome Res* **18**, 923-933 (2019).
3. Ouidir T, Jarnier F, Cosette P, Jouenne T, Hardouin J. Extracellular Ser/Thr/Tyr phosphorylated proteins of *Pseudomonas aeruginosa* PA14 strain. *Proteomics* **14**, 2017-2030 (2014).
4. Gaviard C, Broutin I, Cosette P, De E, Jouenne T, Hardouin J. Lysine succinylation and acetylation in *Pseudomonas aeruginosa*. *J Proteome Res* **17**, 2449-2459 (2018).
5. Receveur-Brechot V, Durand D. How random are intrinsically disordered proteins? A small angle scattering perspective. *Curr Protein Pept Sci* **13**, 55-75 (2012).
6. Wong E*, et al.* The *Vibrio cholerae* colonization factor GbpA possesses a modular structure that governs binding to different host surfaces. *PLoS Pathog* **8**, e1002373 (2012).
7. Nunn MA*, et al.* Complement inhibitor of C5 activation from the soft tick *Ornithodoros moubata*. *J Immunol* **174**, 2084-2091 (2005).
8. Han X, He L, Xin L, Shan B, Ma B. PeaksPTM: Mass spectrometry-based identification of peptides with unspecified modifications. *J Proteome Res* **10**, 2930-2936 (2011).
9. Pernot P*, et al.* Upgraded ESRF BM29 beamline for SAXS on macromolecules in solution. *J Synchrotron Radiat* **20**, 660-664 (2013).
10. Gasteiger E*, et al.* Protein identification and analysis tools on the ExPASy server. In: *The Proteomics Protocols Handbook* (ed Walker JM). Humana Press (2005).
11. Whitten AE, Cai S, Trewhella J. *MULCh* : modules for the analysis of small-angle neutron contrast variation data from biomolecular assemblies. *J Appl Cryst* **41**, 222-226 (2008).

12. Franke D, *et al. ATSAS 2.8*: a comprehensive data analysis suite for small-angle scattering from macromolecular solutions. *J Appl Crystallogr* **50**, 1212-1225 (2017).
13. Konarev PV, Volkov VV, Sokolova AV, Koch MHJ, Svergun DI. *PRIMUS* : a Windows PC-based system for small-angle scattering data analysis. *J Appl Cryst* **36**, 1277-1282 (2003).
14. Franke D, Svergun DI. *DAMMIF*, a program for rapid *ab-initio* shape determination in small-angle scattering. *J Appl Crystallogr* **42**, 342-346 (2009).
15. Volkov VV, Svergun DI. Uniqueness of *ab initio* shape determination in small-angle scattering. *J Appl Crystallogr* **36**, 860-864 (2003).
16. Svergun DI. Restoring low resolution structure of biological macromolecules from solution scattering using simulated annealing. *Biophys J* **76**, 2879-2886 (1999).
17. Grudinin S, Garkavenko M, Kazennov A. *Pepsi-SAXS*: an adaptive method for rapid and accurate computation of small-angle X-ray scattering profiles. *Acta Crystallogr D Struct Biol* **73**, 449-464 (2017).
18. Delvillani F*, et al.* Tet-Trap, a genetic approach to the identification of bacterial RNA thermometers: application to *Pseudomonas aeruginosa*. *RNA* **20**, 1963-1976 (2014).
19. Edgar RC. MUSCLE: multiple sequence alignment with high accuracy and high throughput. *Nucleic Acids Res* **32**, 1792-1797 (2004).
20. Robert X, Gouet P. Deciphering key features in protein structures with the new ENDscript server. *Nucleic Acids Res* **42**, W320-324 (2014).
21. Huerta-Cepas J, Serra F, Bork P. ETE 3: Reconstruction, analysis, and visualization of phylogenomic data. *Mol Biol Evol* **33**, 1635-1638 (2016).
22. Holm L. Benchmarking fold detection by DaliLite v.5. *Bioinformatics* **35**, 5326-5327 (2019).
23. Aleshin AE, DiScipio RG, Stec B, Liddington RC. Crystal structure of C5b-6 suggests structural basis for priming assembly of the membrane attack complex. *J Biol Chem* **287**, 19642-19652 (2012).
