# Supplementary material for: The lytic polysaccharide monooxygenase CbpD promotes Pseudomonas aeruginosa virulence in systemic infection
Source: Nat Commun. 2021 Feb 23;12:1230. doi: 10.1038/s41467-021-21473-0 (PMC7902821; doi:10.1038/s41467-021-21473-0)
Supplement: Supplementary file 3 — Description of Additional Supplementary Files [file 41467_2021_21473_MOESM3_ESM.docx]

**Description of Additional Supplementary Files**

**File Name: Supplementary Data 1**

**Description:** The data provided show the binding of fluorescently labeled CbpD purified from either *Pseudomonas aerugionsa* (rCbpD_PA_) or *E. coli* (rCbpD_EC_) to version 5.3 of the mammalian glycan array provided by the National Consortium for Functional Glycomics (NCFG). Monosaccharides are named according to the established IUPAC nomenclature. The "Sp*n*" ending of all glycan names indicates the spacer type used for attaching the glycan to the glycan chip surface. The "a" or "b" following the monosaccharide abbreviation indicates α− or β-configuration, of the anomeric carbon, respectively. Detailed information can be found at the NCFG website ([www.functionalglycomics.org](http://www.functionalglycomics.org)). The screening was performed in 15 mM Tris-HCl pH 7.5, 150 mM NaCl at final rCbpD concentrations of 5 or 50 µg.ml^-1^. Data are presented as mean ± standard deviation (SD) of one experiment performed in triplicate.

**File Name: Supplementary Data 2**

**Description:** List of identified proteins from the WT and ΔCbpD proteomes obtained from growth in LB, RPMI or RPMI/NHS. The results are obtained from three biological repeats and the data are presented as log_2_ LFQ values.

**File Name: Supplementary Data 3**

**Description:** List of differentially regulated proteins and *q* values of significance identified by comparing the ΔCbpD versus WT proteomes upon growth in LB, RPMI or RPMI/NHS. Cut off values for significance were set to fold change (FC) ≥ 1.5 (log_2_=0.58) and *q* ≤ 0.05 (-log_10_=1.3) in a paired two-tailed *t-*test. Proteins associated with virulence properties are marked in bold.

**File Name: Supplementary Data 4**

**Description:** List of identified proteins from the PA14 and ΔCbpD proteomes obtained from growth in LB, RPMI or RPMI/NHS, only detected in one of the strains, but not in both. These proteins were included in the hypergeometric enrichment analysis together with Supplementary Data 3. When present or absent in ΔCbpD compared to PA14, these proteins were classified as up or down regulated and do not have an associated fold change or *q* value.

**File Name: Supplementary Data 5**

**Description:** Protein clusters obtained by hierarchical clustering of proteins expressed by PA14 and ΔCbpD in three different growth conditions (LB, RPMI and RPMI-NHS).

**File Name: Supplementary Data 6**

**Description:** List of commonly or uniquely identified proteins from the PA14 and ΔCbpD proteomes obtained from growth in LB, RPMI or RPMI/NHS. The list covers differentially regulated proteins (Supplementary Data 3), or proteins only detected in one of the strains but not in both (Supplementary Data 4).

**File Name: Supplementary Data 7**

**Description:** Spleen proteome of PA14, ΔCbpD- and mock-infected mice. The results are presented as log_2_ LFQ.

**File Name: Supplementary Data 8**

**Description:** List of differentially regulated proteins identified by comparing the PA14 (n=8)/ΔCbpD (n=7)-infected versus mock-infected (control, n=4) proteomes. Cut off values for significance were set to fold change (FC) ≥ 1.5 (log_2_=0.58) and *q* ≤ 0.05 (-log_10_=1.3) in a two-tailed paired *t-*test.

**File Name: Supplementary Data 9**

**Description:** List of identified proteins from the PA14/ΔCbpD-infected versus mock-infected (control) proteomes, only detected in infected or mock-infected, but not in both. These proteins were included in the enrichment analysis together with Supplementary Data 8. When present or absent in ΔCbpD/PA14 compared to control, these proteins were classified as up or down regulated, and do not have an associated fold change or *q* value.

**File Name: Supplementary Data 10**

**Description:** List of commonly or uniquely regulated proteins from the PA14/ΔCbpD-infected versus mock-infected (control) proteomes. The list covers differentially regulated proteins from Supplementary Data 8. Cut off values for significance were set to fold change (FC) ≥ 1.5 (log_2_=0.58) and *q* ≤ 0.05 (-log_10_=1.3) in a two-tailed paired *t-*test.

**File Name: Supplementary Data 11**

**Description:** List of commonly or uniquely regulated proteins from the PA14/ΔCbpD-infected versus mock-infected (control) proteomes. The list cover proteins only detected in infected or mock-infected, but not in both from Supplementary Data 9.
