# Supplementary material for: The lytic polysaccharide monooxygenase CbpD promotes Pseudomonas aeruginosa virulence in systemic infection
Source: Nat Commun. 2021 Feb 23;12:1230. doi: 10.1038/s41467-021-21473-0 (PMC7902821; doi:10.1038/s41467-021-21473-0)
Supplement: Supplementary file 15 — Reporting Summary [file 41467_2021_21473_MOESM15_ESM.pdf]

Corresponding author(s): Gustav Vaaje-Kolstad, Victor Nizet and Fatemeh Askarian

Last updated by author(s): Jan 25, 2021

Reporting Summary

Nature Research wishes to improve the reproducibility of the work that we publish. This form provides structure for consistency and transparency in reporting. For further information on Nature Research policies, see our Editorial Policies and the Editorial Policy Checklist.

Statistics

For all statistical analyses, confirm that the following items are present in the figure legend, table legend, main text, or Methods section.

n/a	Confirmed	
☐	☒	The exact sample size (n) for each experimental group/condition, given as a discrete number and unit of measurement
☐	☒	A statement on whether measurements were taken from distinct samples or whether the same sample was measured repeatedly
☐	☒	The statistical test(s) used AND whether they are one- or two-sided<br>*Only common tests should be described solely by name; describe more complex techniques in the Methods section.*
☒	☐	A description of all covariates tested
☐	☒	A description of any assumptions or corrections, such as tests of normality and adjustment for multiple comparisons
☐	☒	A full description of the statistical parameters including central tendency (e.g. means) or other basic estimates (e.g. regression coefficient) AND variation (e.g. standard deviation) or associated estimates of uncertainty (e.g. confidence intervals)
☐	☒	For null hypothesis testing, the test statistic (e.g. F, t, r) with confidence intervals, effect sizes, degrees of freedom and P value noted<br>*Give P values as exact values whenever suitable.*
☒	☐	For Bayesian analysis, information on the choice of priors and Markov chain Monte Carlo settings
☒	☐	For hierarchical and complex designs, identification of the appropriate level for tests and full reporting of outcomes
☒	☐	Estimates of effect sizes (e.g. Cohen's d, Pearson's r), indicating how they were calculated

Our web collection on statistics for biologists contains articles on many of the points above.

Software and code

Policy information about availability of computer code

Data collection

Proteomics data collection: Xcalibur 2.2 SP1, HPLC data collection: Chromeleon v7.2.9, SAXS data collection: BsxCUBE, Microscope Slide scanning: MicroVisioneer, Fluorescence imaging: ZEN Blue, TEM: Philips CM10 microscope.

Data analysis

General data analysis: Microsoft Excel for Office 365 MSO (16.0.12527.21378)

HPLC data analysis:
Chromeleon v7.2.9,

Proteomics analysis:
MaxQuant v1.6.3.3
PEAKS (for PTM analysis)
Metascape (Stable release 3.5,1 March 2019, Zhou et al. Nature Commun. 2019 10(1):1523)
String App for Cytoscape v3.7.1
QIAGEN Ingenuity Pathway Analysis (IPA) (QIAGEN Inc.)

Homology modelling and simulation:
RaptorX 2020 version (online template-based protein structure modeling server)
Aber v18
AmberTools v19
H++ v1.0

Protein sequence analysis:

PhyML (v20160115)
ESPript (v3.0)

SAXS analysis:
ATSAS v3.0 (sub-programs ALMERGE, PRIMUS, DAMMIF, DAMAVER, DAMMIN and SUPCOMB)
Pepsi-SAXS v2.6

Protein structure graphics:
PyMol 2.3.4.

Statistical analysis:
GraphPad Prism 8.0 (GraphPad Inc., San Diego, CA)
MATLAB R2018b (proteomics data).

FACS:
FloJo v10
Cellstream

Micrograph scanning and analysis:
MicroVisioneer

Fluorescence microscopy imaging and analysis:
ImageJ/Fiji v 2.1.0/1.53c

For manuscripts utilizing custom algorithms or software that are central to the research but not yet described in published literature, software must be made available to editors and reviewers. We strongly encourage code deposition in a community repository (e.g. GitHub). See the Nature Research guidelines for submitting code & software for further information.

Data

Policy information about availability of data

All manuscripts must include a data availability statement. This statement should provide the following information, where applicable:

- Accession codes, unique identifiers, or web links for publicly available datasets
- A list of figures that have associated raw data
- A description of any restrictions on data availability

The constructs generated in this study are available upon request. Source data are provided with this paper. The mass spectrometry proteomics data have been deposited to the ProteomeXchange Consortium via the PRIDE 134 partner repository with dataset identifiers PXD017971, PXD021888, PDX018769. SAXS data have been deposited at the SASBDB (SASBDB IDs: SASDK42 (20 °C), SASDJQ5 (24 °C), SASDJR5 (37 °C). The CbpD structure prediction was based on templates provided by publicly available protein structures with PDB identifiers 6IF7, 2XWX, 2I1S. 4TX8, 1ED7, 2D49, 5IN1 and 4XZJ. For analysis of CbpD structural similarity the following publicly available structures were used: PDB identifiers 2XWX (for comparison with GbpA) and 4A5W (for comparison with the C5bC6-complex). All data required to evaluate the paper's conclusions are present in the paper, the Supplementary information, data sets and source data. Any additional data are available from the corresponding authors upon reasonable request.

Field-specific reporting

Please select the one below that is the best fit for your research. If you are not sure, read the appropriate sections before making your selection.

☒ Life sciences ☐ Behavioural & social sciences ☐ Ecological, evolutionary & environmental sciences

For a reference copy of the document with all sections, see nature.com/documents/nr-reporting-summary-flat.pdf

Life sciences study design

All studies must disclose on these points even when the disclosure is negative.

Sample size	No statistical pre-determination of sample size was performed. Sample sizes selected were based on results from pilot experiments or based on other published work that gave reliable statistical results.
Data exclusions	No data were excluded from the analyses with one exception: To identify the organ-specific proteome responses to PA14 and △CbpD infection, one out of the eight independent biological replicates in △CbpD-infected mice showed only 10% of the total identified protein IDs compared to other (n=7) independent biological replicates. Our additional analysis revealed a low Pearson correlation coefficient value for this sample compared to the rest of the replicates under △CbpD-infection treatment. Thus this sample was considered as an outlier and omitted from the analysis.
Replication	All attempts at replication were successful. The number of experimental replicates are indicated in the respective figure legends.
Randomization	For the in vivo infection model studies, mice were randomly distributed/grouped from housing cages to experimental cages that comprised control and treatment (different infection conditions) cages. Randomization was not relevant for the other experiments performed in this

study.

Blinding

In general, blinding was considered unnecessary for the experiments associated with this study as prior knowledge was required to carry out experiments and analysis. The only exception was the histopathological analysis, where slide evaluation and scoring was performed blindly.

Reporting for specific materials, systems and methods

We require information from authors about some types of materials, experimental systems and methods used in many studies. Here, indicate whether each material, system or method listed is relevant to your study. If you are not sure if a list item applies to your research, read the appropriate section before selecting a response.

Materials & experimental systems

n/a	Involved in the study	
☐	☒	Antibodies
☐	☒	Eukaryotic cell lines
☒	☐	Palaeontology and archaeology
☐	☒	Animals and other organisms
☐	☒	Human research participants
☒	☐	Clinical data
☒	☐	Dual use research of concern

Methods

n/a	Involved in the study	
☒	☐	ChIP-seq
☐	☒	Flow cytometry
☒	☐	MRI-based neuroimaging

Antibodies

Antibodies used

A custom made affinity-purified CbpD antibody was generated by Davids Biotechnologie by immunizing rabbits with peptides KDGYNPEKPLAWSDLEPA and DAQGRDAQRHSLTLAQGANGA.

The commercially used antibodies that were described in the "Methods" are listed as follow:

BD Biosciences: Phycoerythrin (PE)–conjugated monoclonal antibodies (mAbs) directed against:
CD114 (PE mouse anti-human CD114, LMM741, Cat no. 554538, dilution: 1:10), CD87 (PE mouse anti-human CD87, VIM5, Cat no. 555768, dilution 1:3), CD54 (PE mouse anti-human CD54, HA58, Cat no. 555511, dilution 1:7.5), CD58 (PE mouse anti-human CD58, 1C3, Cat no. 555921, dilution 1:10), CD35 (PE mouse anti-human CD35, E11, Cat no. 559872, dilution 1:6), CD47 (PE mouse anti-human CD47, B6H12, Cat no. 556046, dilution 1:5), CD119 (PE mouse anti-human CD119, GIR-208, Cat no. 558934, dilution 1:15), CD49b (PE mouse anti-human CD49b, 12F1, Cat no. 555669, dilution 1:15), and CD44 (PE mouse anti-human CD44, 515, Cat no. 550989, dilution 1:150).

BD Biosciences: Fluorescein isothiocyanate (FITC)–labeled mAbs directed against CD11a (FITC mouse anti-human CD11a, HI111, Cat no.5553, dilution 1:4), CD15 (Mouse anti-human CD15-FITC, MMA, Cat no.332778, dilution 1:150), CD147 (FITC mouse anti-human CD147, HIM6, Cat no: 555962, dilution: 1:10), CD18 (FITC Mouse Anti-Human CD18, 3G8, Cat no. 347953, dilution 1:15), CD31 (FITC Mouse Anti-Human CD31, WM54, Cat no. 555445, dilution 1:10), CD46 (FITC Mouse Anti-Human CD46, E4.3, Cat no. 555949, dilution 1:15), CD9 (FITC Mouse Anti-Human CD9, M-L13, Cat no. 555371, dilution 1:7.5), and CD66 (B1.1, FITC Mouse Anti-Human CD66, Cat no: 551479, 1:15)

BD Biosciences: Allophycocyanin (APC)–labeled monoclonal antibodies against CD13 (APC mouse anti-human CD13, WM15, Cat no. 557454, dilution 1:4), CD14 (APC mouse anti-human CD14, M5E2, Cat no. 555399, dilution 1:4), CD16 (Alexa Fluor® 647 mouse anti-human CD16 , 3G8, Cat no. 557710, dilution 1:300), CD11b (APC mouse anti-human CD11b, CRF44, Cat no. 550019, dilution 1:4) , CD11c (APC mouse anti-human, B-ly6, Cat no. 559877, dilution 1:4), CD29 (APC mouse anti-human CD29, MAR4, Cat no. 559883, dilution 1:10), CD55 (APC mouse anti-human CD55, IA10, Cat no. 555696, dilution 1:15) and CD45 (APC mouse anti-human CD45, HI30, Cat no. 555485, dilution 1:75)

R&D Systems: CD120a-Alexa Fluor488 (Anti-human TNF RI/TNFRSF1A Alexa Fluor® 488-conjugated antibody, 16803.161, Cat no. FAB225G, dilution 1:4), CD120b-FITC (Anti-human TNF RII/TNFRSF1B fluorescein-conjugated antibody, 22235.311, Cat no. FAB226F, dilution 1:5), CD181-PE (anti-human CXCR1/IL-8RA PE-conjugated Antibody, 42705.111, Cat no. FAB330P, dilution 1:10), CD182-PE (anti-human CXCR2/IL-8RB PE-conjugated Antibody, CXCR2, Cat no. FAB331P, dilution 1:6), and Siglec-9-APC (Human Siglec-9 APC-conjugated antibody, 191240, Cat no. FAB1139A, dilution 1:20)

Beckman Coulter ImmunoTech: CD63-PE (Mouse anti-human CD63-PE, CLB-gran/12, Cat no. IM1914U, dilution 1:15)

Santa Cruz: CD43-FITC (mouse anti-human CD43, 6D269, Cat no. sc-70684 FITC, dilution 1:15), C5b-9 (mouse-anti human C5b-9, aE11, Cat no. sc-58935, Dilution 1: 100=1 µg/ml).

Abd-Serotec (Currently called Bio-Rad): BLTR-FITC (Mouse anti-human BLTR:FITC, 202/7B1, Cat no. MCA2108F, dilution 1:7.5), CD89 (Mouse anti-human CD89, Mip8a, Cat no: MCA1824PE, dilution 1:7.5)

Novus Biologicals: CD32-PE (Mouse anti-Human CD32-PE, 7.3, Cat no. NBP2-47830PE, dilution 1:150).

Ebioscience: CD282-PE (Rat anti-human CD44 (IM7)-PE, T2.5, Cat no. 12-9024-82, dilution: 1:30)

Invitrogen: CD10-APC (Mouse Anti-human CD10 Monoclonal Antibody-APC, MEM-78, Cat no. CD1005, dilution: 1:10), fMLP-FITC (FITC-conjugated formyl-Nle-Leu-Phe-Nle-Tyr-Lys, Cat no. F1314, dilution 1:1000), HRP-conjugated goat anti-rabbit IgG (Goat anti-Rabbit IgG (H+L) secondary antibody-HRP, Cat no: 65-6120, dilution 1:5000), Alexa Fluor488-conjugated goat anti-mouse IgG (Goat anti-Mouse IgG (H+L) cross-adsorbed secondary antibody- Alexa Fluor 488, Cat no. A11001, dilution 1:2000=1 µg/ml)

BioLegend: CD88-PE (Mouse anti anti-human CD88 (C5aR)-PE, S5/1, Cat no. 344304, dilution 1:50)

Quidel: Murine monoclonal anti-human Bb (Murine anti-human Factor B (Bb) Protein, Cat no. A227, final concentration in the assay 1 µg/ml)

Recived as a gift from an individual lab: Mouse-anti C3b (produced and Alexa Fluor 488-labeled in house) have been described in Heesterbeek et al., 2019. EMBO J 38: e99852. Final concentration in the assay 3 µg/ml)

Validation

CbpD antibody recognizes secreted CbpD by Pseudomonas aeruginosa (e.g. Supplementary Fig. 11a). Species reactivity: rat.

The link or information associated with validation of the rest of primary antibodies are as follow:

CD114: https://www.bdbiosciences.com/us/applications/research/stem-cell-research/hematopoietic-stem-cell-markers/human/negative-markers/pe-mouse-anti-human-cd114-lmm741/p/554538

CD87: https://www.bdbiosciences.com/us/reagents/research/antibodies-buffers/immunology-reagents/anti-human-antibodies/cell-surface-antigens/pe-mouse-anti-human-cd87-vim5-also-known-as-vim-5/p/555768

CD54: https://www.bdbiosciences.com/eu/applications/research/t-cell-immunology/regulatory-t-cells/surface-markers/human/pe-mouse-anti-human-cd54-ha58/p/555511

CD58: https://www.bdbiosciences.com/us/reagents/research/antibodies-buffers/immunology-reagents/anti-human-antibodies/cell-surface-antigens/pe-mouse-anti-human-cd58-1c3-also-known-as-aicd586/p/555921

CD35: https://www.bdbiosciences.com/us/applications/research/b-cell-research/surface-markers/human/pe-mouse-anti-human-cd35-e11/p/559872

CD47: https://www.bdbiosciences.com/us/applications/research/stem-cell-research/cancer-research/human/pe-mouse-anti-human-cd47-b6h12/p/556046

CD89: https://www.bio-rad-antibodies.com/monoclonal/human-cd89-antibody-mip8a-mca1824.html

CD119: https://www.bdbiosciences.com/us/applications/research/t-cell-immunology/th-1-cells/surface-markers/human/pe-mouse-anti-human-cd119-gir-208/p/558934

CD49b: https://www.bdbiosciences.com/us/reagents/research/antibodies-buffers/immunology-reagents/anti-human-antibodies/cell-surface-antigens/pe-mouse-anti-human-cd49b-12f1/p/555669

CD11a: https://www.bdbiosciences.com/us/applications/research/t-cell-immunology/regulatory-t-cells/surface-markers/human/fitc-mouse-anti-human-cd11a-hi111/p/555383

CD147: https://www.bdbiosciences.com/us/applications/research/stem-cell-research/mesoderm-markers/human/fitc-mouse-anti-human-cd147-him6/p/555962

CD15: https://www.bdbiosciences.com/eu/reagents/clinical/reagents/single-antibodies/cd15-fitc-mma/p/332778

CD18: https://www.bdbiosciences.com/us/applications/research/stem-cell-research/mesoderm-markers/human/fitc-mouse-anti-human-cd18-l130/p/347953

Recived as a gift from an individual lab: Mouse-anti C3b (produced and Alexa Fluor 488-labeled in house) have been described and validated in Heesterbeek et al., 2019. EMBO J 38: e99852 and validated in several studies.

CD31: https://www.bdbiosciences.com/us/applications/research/stem-cell-research/cancer-research/human/fitc-mouse-anti-human-cd31-wm59-also-known-as-wm-59/p/555445

CD46: https://www.bdbiosciences.com/us/applications/research/t-cell-immunology/regulatory-t-cells/surface-markers/human/fitc-mouse-anti-human-cd46-e43/p/555949

CD9: https://www.bdbiosciences.com/us/applications/research/stem-cell-research/pluripotent-stem-cell-markers-esc-and-ipsc/human/fitc-mouse-anti-human-cd9-m-l13/p/555371

CD66: https://www.bdbiosciences.com/us/applications/research/stem-cell-research/cancer-research/human/fitc-mouse-anti-human-cd66-b11cd66/p/551479

CD13: https://www.bdbiosciences.com/us/applications/research/stem-cell-research/hematopoietic-stem-cell-markers/human/negative-markers/apc-mouse-anti-human-cd13-wm15/p/557454

CD14: https://www.bdbiosciences.com/eu/applications/research/stem-cell-research/hematopoietic-stem-cell-markers/human/negative-markers/apc-mouse-anti-human-cd14-m5e2/p/555399

CD16: https://www.bdbiosciences.com/us/applications/research/stem-cell-research/cancer-research/human/alexa-fluor-647-mouse-anti-human-cd16-3g8/p/557710

CD11b: https://www.bdbiosciences.com/eu/applications/research/stem-cell-research/mesenchymal-stem-cell-markers-bone-marrow/human/negative-markers/apc-mouse-anti-human-cd11b-icrf44-also-known-as-44/p/550019

CD29: https://www.bdbiosciences.com/us/applications/research/stem-cell-research/pluripotent-stem-cell-markers-esc-and-ipsc/human/apc-mouse-anti-human-cd29-mar4/p/559883

CD45: https://www.bdbiosciences.com/eu/applications/research/stem-cell-research/cancer-research/human/apc-mouse-anti-human-cd45-hi30/p/555485

CD55: https://www.bdbiosciences.com/us/applications/research/stem-cell-research/mesenchymal-stem-cell-markers-adipose/human/positive-markers/apc-mouse-anti-human-cd55-ia10/p/555696

CD120a: https://www.bio-techne.com/p/antibodies/human-tnf-ri-tnfrsf1a-alexa-fluor-488-conjugated-antibody-16803_fab225g

CD120b: https://www.bio-techne.com/p/antibodies/human-tnf-rii-tnfrsf1b-fluorescein-conjugated-antibody-22235_fab226f

CD181: https://www.bio-techne.com/p/antibodies/human-cxcr1-il-8ra-pe-conjugated-antibody-42705_fab330p

CD182: https://www.bio-techne.com/p/antibodies/human-cxcr2-il-8rb-pe-conjugated-antibody-48311_fab331p

Siglec-9: https://www.bio-techne.com/p/antibodies/human-siglec-9-apc-conjugated-antibody-191240_fab1139a

CD63: https://www.beckman.com/reagents/coulter-flow-cytometry/antibodies-and-kits/single-color-antibodies/cd63/im1914u

CD43: https://datasheets.scbt.com/sc-70684.pdf

C5b-9 (aE11): https://www.scbt.com/p/c5b-9-antibody-ae11

BLTR: https://www.bio-rad-antibodies.com/static/datasheets/mca21/human-bltr-antibody-202-7b1-mca2108f.pdf

CD89: https://www.bio-rad-antibodies.com/static/datasheets/mca18/human-cd89-antibody-mip8a-mca1824pe.pdf

CD32: https://www.novusbio.com/products/fc-gamma-riia-cd32a-antibody-73_nbp2-47830pe?utm_source=distributor&utm_medium=referral&utm_campaign=product#datasheet

CD282: https://www.thermofisher.com/antibody/product/CD44-Antibody-clone-IM7-Monoclonal/12-0441-82

CD10: https://www.thermofisher.com/antibody/product/CD10-Antibody-clone-MEM-78-Monoclonal/CD1005

CD88: https://www.biolegend.com/en-us/products/pe-anti-human-cd88-c5ar-antibody-6162

Factor Bb: https://www.quidel.com/research/monoclonal-antibodies/monoclonal-antibody-to-human-factor-BBb

FITC-fMLP: https://www.thermofisher.com/order/catalog/product/F1314?us&en

Recived as a gift from an individual lab: Mouse-anti C3b (produced and Alexa Fluor 488-labeled in house) have been described and validated in Heesterbeek et al., 2019. EMBO J 38: e99852 , while used in another studies (e.g., Doordujin et al., 2020. PLoS Pathog 16 (6): e1008606).

Eukaryotic cell lines

Policy information about cell lines

Cell line source(s)	(THP-1) and HL-60 cell lines were purchased from ATCC

Authentication	Verified by the supplier (ATCC) at the time of distribution via a series of morphology, karyotyping, and PCR based approaches (e.g., growth curves, cytochrome C oxidase I gene (COI) analysis and short tandem repeat (STR) profiling)
Mycoplasma contamination	Cell lines were negative to mycoplasma (tested and verified by ATCC)
Commonly misidentified lines (See ICLAC register)	No commonly misidentified cell lines were used in this study.

Animals and other organisms

Policy information about studies involving animals; ARRIVE guidelines recommended for reporting animal research

Laboratory animals	Mus Musculus (strain CD-1 mice), eight-week-old female, kept in filter-top cages with access to food pellet and water under controlled ambient temperature (20-22 °C) and relative humidity (30-70%), 12h light/12h dark cycle
Wild animals	No wild animal were used in this study
Field-collected samples	No field-collected samples were used in the study.
Ethics oversight	As described in the "Ethics Declarations" section, animal experiments/housing were conducted under the UC San Diego approved IRB protocol S00227M, and in accordance with the rules and regulations of the Institutional Animal Care and Use Committee.

Note that full information on the approval of the study protocol must also be provided in the manuscript.

Human research participants

Policy information about studies involving human research participants

Population characteristics	The participant were healthy individuals, comprised of both male and female, age range 20-50 and from different ethnic groups
Recruitment	Any healthy male and female were eligible to voluntarily participation and were recruited under the protocols that has been approved by UMC Medical Ethics Committee and by REK-Norway (2018/1586). The blood donors provided written informed consent and there were no known biases that contributed towards their study inclusion other than that they were healthy and working, studying or visiting the same institution where the experiment was conducted.
Ethics oversight	As described in the "Ethics Declarations" section: "Blood was drawn from several healthy volunteers (male and female) in accordance with ethical principles of the Helsinki Declaration and under the protocols that has been approved by UMC Medical Ethics Committee and REK-Norway (2018/1586). All blood donors provided written informed consent."

Note that full information on the approval of the study protocol must also be provided in the manuscript.

Flow Cytometry

Plots

Confirm that:

- [x] The axis labels state the marker and fluorochrome used (e.g. CD4-FITC).
- [x] The axis scales are clearly visible. Include numbers along axes only for bottom left plot of group (a 'group' is an analysis of identical markers).
- [x] All plots are contour plots with outliers or pseudocolor plots.
- [x] A numerical value for number of cells or percentage (with statistics) is provided.

Methodology

Sample preparation	Flow cytomteric analysis was performed on bacteria monoculture or immune cells (with focus on PMNs) obtained from blood . Sample preparation is described in detail in the "Methods" under sections: "Complement deposition on bacteria surface", "Whole-blood phagocytosis", and "Competition for surface-expressed receptor binding".
Instrument	CellStream (Luminex)<br>MACSQuant (Miltenyi biotech)<br>FACSVerse (Becton Dickinson)
Software	FlowJo and CellStream software
Cell population abundance	10000 events per condition (from gated cells)
Gating strategy	Gating of cells was carried out on the basis of forward and side scatter. The fluorescence intensity (FL) of 10,000 gated neutrophils/bacteria was measured for each sample. Phagocytosis/binding of antibody to the surface expressed receptors/ deposition of complement components on the bacterial surface (C3b, Bb, C5b9) was identified when neutrophils/bacteria

expressed fluorescence. The geometric mean of the fluorescence intensity (GMFI) of the gated cells was calculated using CellStream software/FlowJo. Figures exemplifying the gating strategy and fluorescence response of the cells are provided in the supplementary information (Supplemental Figure 16 and 17).

☒ Tick this box to confirm that a figure exemplifying the gating strategy is provided in the Supplementary Information.
